# Luzp4 defines a new mRNA export pathway in cancer cells

**DOI:** 10.1093/nar/gkv070

**Published:** 2015-02-06

**Authors:** Nicolas Viphakone, Marcus G. Cumberbatch, Michaela J. Livingstone, Paul R. Heath, Mark J. Dickman, James W. Catto, Stuart A. Wilson

**Affiliations:** 1 Department of Molecular Biology and Biotechnology, The University of Sheffield, Firth Court, Western Bank, Sheffield, UK; 2 Academic Urology Unit, The University of Sheffield, Beech Hill Road, Sheffield, UK; 3 Sheffield Institute for Translational Neuroscience, The University of Sheffield, 385a Glossop Road, Sheffield, UK; 4 Department of Chemical and Biological Engineering, The University of Sheffield, Mappin Street, Sheffield, UK

## Abstract

Cancer testis antigens (CTAs) represented a poorly characterized group of proteins whose expression is normally restricted to testis but are frequently up-regulated in cancer cells. Here we show that one CTA, Luzp4, is an mRNA export adaptor. It associates with the TREX mRNA export complex subunit Uap56 and harbours a Uap56 binding motif, conserved in other mRNA export adaptors. Luzp4 binds the principal mRNA export receptor Nxf1, enhances its RNA binding activity and complements Alyref knockdown *in vivo*. Whilst Luzp4 is up-regulated in a range of tumours, it appears preferentially expressed in melanoma cells where it is required for growth.

## INTRODUCTION

The transport of mRNA from the nucleus to the cytoplasm is an essential step in eukaryotic gene expression. It is subject to rigorous quality control to ensure that only fully processed mature mRNA exits the nucleus (1). A major mRNA export pathway used in human cells involves the multisubunit TREX complex which comprises the THO complex and numerous additional subunits (2,3). TREX integrates information from transcription (4), splicing (5) and 3′ end processing (6,7) to mark mature mRNAs for export. TREX assembly requires Alyref, the THO complex, and is driven by the Uap56 RNA helicase (3,8). A number of TREX subunits including Alyref, Chtop and Uif harbour a specific peptide motif (Uap56 binding motif, UBM) that allows direct interaction with Uap56 or its paralogue, Ddx39 (9,10).

Nxf1 is the major metazoan mRNA export receptor which functions as a heterodimer with Nxt1 (11). Free Nxf1 sequesters its own N-terminal RNA binding domain but upon binding to TREX, the RNA binding domain of Nxf1 is exposed allowing direct interactions with the mRNA (12,13). The key TREX subunits responsible for this conformational change in Nxf1 (to allow mRNA binding) are Alyref, which binds the N-terminal region of Nxf1, and Thoc5 or Chtop, either of which can bind the NTF2-like (NTF2L) domain (aa 371-550) (10,13). A number of other proteins that function in a manner similar to Alyref in binding Nxf1 have been identified, including Uif, various SR proteins and U2af1 (9,14–16). These interactions probably ensure that multiple Nxf1 molecules are recruited to a single mRNA, ensuring efficient translocation of the mRNP across the nuclear pore. Whilst Nxf1 is the major mRNA export receptor, there exist several other *Nxf* genes in humans (17) including *Nxf2*. Notably, Nxf2 destabilizes Nxf1 mRNA in testis and neurons and thus Nxf2 may represent the major mRNA export receptor in these tissues (18).

There is increasing evidence that various proteins involved in mRNA export are dysregulated in cancer cells (19). These proteins include eIF4E which is up-regulated in ∼30% of cancer cells. eIF4E is required for the export of specific transcripts via the CRM1 pathway and also remodels the nuclear pore complex which promotes mRNA export and oncogenic transformation (20). The TREX subunit Thoc5 is the target of leukaemogenic tyrosine kinases (21) and two other TREX subunits, Thoc1 (Hpr1) and Alyref, are dysregulated in cancer cells (22–24). Furthermore, the correct packaging of mRNA by TREX is important to prevent R-loop formation and in TREX mutants, increased R-loop formation leads to genome instability (24). The TREX-2 complex, which is implicated in the transfer of mRNA from the nuclear interior to the nuclear pore complex (25), has also been shown to be associated with BRCA-2 and is implicated in maintaining genome stability (26). Together these studies highlight the close link between packaging/export of mRNA and genome stability/cancer.

One important class of proteins implicated in cancer are the cancer testis antigens (CTAs), which are a diverse collection of proteins characterized by their largely restricted expression in testis in the normal situation and up-regulation in one or more cancers. Many CTAs reside on the X-chromosome and a key element in their induction in cancer cells appears to be promoter demethylation (27). The unique expression characteristics of CTAs means they represent a promising group of targets both as biomarkers and therapeutic targets in the treatment of cancer. However, despite the identification of a wide variety of CTAs, in general the molecular functions of this class of proteins are poorly characterized though Nxf2 is classified as a CTA (28). Here, we show that the CTA Luzp4 is an mRNA export factor required for efficient growth of melanoma cells.

## MATERIALS AND METHODS

### Plasmids, antibodies and cell culture

Plasmids encoding CBP80-Myc, MS2-GFP, MS2-Alyref, MS2-Nxf1, pLUCSALRRE6MS2, GST-Uap56, GB1-Nxf1 and truncations are described previously (12) as were FLAG-Nxf1-myc, FLAG-GFP, FLAG-Srsf1-myc, GST-Nxf1:p15 (13). FLAG-Uap56-myc was generated by subcloning the Uap56 open reading frame into p3X-FLAG-Myc-CMV26 (Sigma). Luzp4 cDNA (NM_016383) used for all constructs was purchased from Source Bioscience (clone MGC:149218, IMAGE:40112435, locus BC128134). All Luzp4 constructs (wild-type or mutant) used for *in vitro* pulldowns and UV-crosslinking experiments were expressed as 6-His tagged fusions from pET24b (cloned *Nde*I/*Xho*I as PCR products). FLAG-Luzp4 and Luzp4-myc used in co-immunoprecipitation (Co-IP) experiments were expressed from p3XFLAG-myc-CMV26 (cloned *Hind*III/*Eco*RI) and pcDNA3.1-myc-HisB (cloned *Bam*HI/*Xho*I), respectively. To make the FLAG-Luzp4 stable cell line, 3XFLAG-Luzp4 was amplified by PCR from p3XFLAG-Luzp4-myc (see above) and cloned *Not*I/*Xho*I into a modified pcDNA5-FRT-TO-His where the start codon of the N-terminal 6His tag was destroyed by site-directed mutagenesis. The cell line was generated as previously described (9). A vector pEGFPN1-Luzp4 (*Xho*I/*Bam*HI) was built to monitor GFP-Luzp4 localisation in HeLa cells. This vector was used as a template to generate the deletion mutant constructs pEGFPN1-Luzp4(ΔN) (amino acids 119–313), pEGFPN1-Luzp4(Δ aa 22–40), pEGFPN1-Luzp4(Δ aa 156–236), pEGFPN1-Luzp4(Δ aa 156–178) by divergent PCR. pEGFPN1-luzp4(ΔC) was made by amplifying Luzp4 region encoding aa 1–240 by PCR and cloning the resulting PCR product into pEGFPN1 (*Xho*I/*Bam*HI). For MS2 mRNA export assays, Luzp4 was amplified by PCR as an *Nhe*I/*Not*I product and cloned in pCINeo-MS2 (*Xba*I/*Not*I), described previously (9). Antibodies were obtained from the following suppliers Thoc1 (Hpr1), Nxf1 and Alyref (Abcam); Ars2 (Santa Cruz); Hnrnpa1 (Millipore); FLAG, Tubulin (Sigma); GFP (Clontech). The Thoc5 and Uap56 antibodies have been described previously (13). A rabbit polyclonal antibody was raised against GST-luzp4(ΔN) (expressed from pGEX6P1-Luzp4(aa 119–313)) and used for western analysis (Figure 5 and Supplementary Figure S8B). Cell lines used in functional complementation experiments were built using the strategy depicted in Supplementary Figure S6B and described previously (10), resulting in the expression of Luzp4 (or its mutant versions) with EmGFP fused to its C-terminus. miRNAs hairpins also expressed from these constructs and targeting Alyref and/or Uif were from (9). Growth curves were performed as described previously (9). To determine statistical differences in growth between the various cell lines examined, we first log_10_-transformed the number of cells obtained for each cell line from Day 4 (when the curves start to scatter) for each independent experiment. The slopes of the linear regressions applied to these values were compiled and these growth rates were then compared by a one-way ANOVA analysis followed by a Tukey test.

### siRNA treatments and colony formation assays

For each RNAi condition, three wells of 6-well plates with 50 000 cells/well were transfected twice with 40 nM of the indicated siRNAs and 12 μl Interferin (PolyPlus-transfection^®^)/well (renewed at 48 h). Control siRNA, targeting dsRED sequence, and ALYREF siRNA were 5′-CACCGUGAAGCUGAAGGUG-3′ and 5′-GGAACUCUUUGCUGAAUUU-3′, respectively (7). Uif knockdown was performed with a pool of two siRNAs purchased from Dharmacon: 5′-ACAUAAACAGUGUCGGAAA-3′, 5′-GCAAAGAGAACUCGUCAAU-3′. Luzp4 knockdown was performed with a pool of two siRNAs: 5′-GCCUUCAAGACAGCAAUCA-3′, 5′- CAGAAGGAAAUCCGGACAA-3′. At 60–65 h post transfection (seeding day), each well was phosphate buffered saline (PBS) washed and treated with 100 μl trypsin. All three wells of each RNAi condition were then inactivated and pooled with 2 ml of serum-containing medium. Cell suspensions were homogenized, counted and diluted to 200 cells/ml.

For each RNAi condition, a new 6-well plate was seeded at 200 cells/well, therefore representing six replicates/condition. Each well was adjusted to 2 ml of culture medium and left to grow at 37°C for 10 (293T cells) to 14 days (MeWo cells). On the counting day, cells were washed once with PBS and stained 5 min with 1 ml of crystal violet (0.5 % giemsa powder in methanol) / well. Each well was then washed twice with 1 ml of deionized water and left to dry for at least 15 min. The entire experiment was repeated five times independently and only colonies containing at least 50 cells were counted. One-way ANOVA analysis followed by a Tukey test was performed to compare the effect of each siRNA-treatment on MeWo and 293T cellular proliferation in Figure 6D and Supplementary Figure S8B, respectively.

### Luzp4–6His protein expression and purification

pET24b-Luzp4–6His was transformed into Rosetta2 cells. Five litres of 37°C pre-warmed terrific broth medium (50 μg/ml Kanamycin) was inoculated from a 100 ml overnight culture (terrific broth, 50 μg/ml Kanamycin, 34 μg/ml Chloramphenicol) to give a starting OD_600_ of 0.05. The cells were grown at 37°C up to OD_600_ = 0.5. Production of Luzp4–6His was then induced with 0.5 mM IPTG for three and a half hours at 37°C. Cells were harvested and lysed by sonication on ice in binding buffer (50 mM Tris-HCl pH 8, 1 M NaCl, 5 mM imidazol, 0.5% Triton-X100, 5% Glycerol, 1 mM PMSF, 1X SigmaFAST™ Protease Inhibitors). The lysate was cleared by centrifugation at 30 000xg for 30 min at 4°C. The pellet of insoluble material was washed twice with binding buffer and resuspended in 100 ml of denaturation buffer (50 mM Tris-HCl pH 8, 500 mM NaCl, 8 M Urea) for 20 min at room temperature. Denatured material was first cleared by centrifugation and then incubated with 5 ml of Cobalt beads (TALON^®^) for 30 min. The column was then washed three times with 20 ml of wash buffer (50 mM Tris-HCl pH 8, 1 M NaCl, 5 mM imidazole, 8 M Urea). Luzp4–6His was eluted with 6 × 5 ml of elution buffer (50 mM Tris-HCl pH 8, 1 M NaCl, 200 mM imidazol, 8 M Urea). Glycerol and ethylenediaminetetraacetic acid (EDTA) were immediately added to the pooled fractions at 10% and 1 mM final, respectively. Finally, the purified protein was immediately dialysed twice against 5 l of dialysis buffer (50 mM Tris-HCl pH 7.5, 0.5 M NaCl, 0.05% NP40, 50 mM L-Arginine, 50 mM L-Glutamate, 10% glycerol). In total, ∼40 mg of soluble Luzp4–6His protein was obtained and kept frozen at −80°C as aliquots. GB1-Alyref-6His was produced and purified as described previously (10).

### GST-pulldown experiments and Co-IP

GST-pulldown experiments and Co-IP experiments were carried out as described previously (10,13) using RB100 buffer for GST pulldowns (25 mM HEPES pH 7.5, 100 mM KOAc, 10 mM MgCl_2_, 1 mM dithiothreitol, 0.05% Triton X-100, 10% glycerol, 10 μg/ml RNase A) and IP lysis buffer for Co-IPs (50 mM HEPES pH 7.5, 100 mM NaCl, 1 mM EDTA, 1 mM dithiothreitol, 0.5% Triton X-100 and 10% glycerol + (10 μg/ml RNase A) as indicated in figures). The mRNP capture assays were carried out as described previously (13).

### Mass spectrometry analysis

For mass spectrometry analysis, 20 × 15 cm dishes of the Flp-In™ T-REx™ 293 FLAG-Luzp4 or control Flp-In™ T-REx™ 293 FLAG stable cell lines were induced for 48 h with 1 μg/ml of tetracycline and used for the IP with anti-FLAG MS2-agarose beads (Sigma). Interacting partners were eluted using 1 M Arginine-HCl (pH 3.5) and subsequently brought to pH 7.5 using 1.5 M Tris-HCl pH 8.8. In solution and in gel tryptic digestions were performed as previously described (29,30). Tryptic digests were resuspended in 0.1% final concentration of Trifluoroacetic acid (TFA). Five microlitres was used for LC-MS/MS analysis. Peptides were separated using an Ultimate 3000 RSLC nano liquid chromatography system (ThermoFisher, UK), using a 150 mm × 75 μm I.D. PepMap reversed phase column (ThermoFisher, UK). Linear gradient elution was performed from 95% buffer A (0.1% formic acid) to 40% buffer B (0.1% formic acid, 95% acetonitrile) at a flow rate of 300 nl/min in 60 min. MS/MS analysis was performed using a maXis UHR TOF mass spectrometer (Bruker Daltonics) using an automated acquisition approach. MS and MS/MS scans (m/z 50–2000) were acquired in positive ion mode. Lock mass calibration was performed using HP 1221.990364. Line spectra data were then processed into peak list by Data Analysis (Bruker Daltonics) using the following settings. The sum peak finder algorithm was used for peak detection using a signal-to-noise ratio of 10, a relative to base peak intensity of 0.1% and an absolute intensity threshold of 100. Spectra were deconvoluted and the peak lists exported as Mascot Generic Files (MGF) and searched using Mascot 2.2 server (Matrix Science) The Swiss-Prot database (Swiss-Prot Release 10.5, 20 April 2010, 516 604 sequences) was searched using the following parameters (analysis peptide tolerance = ± 0.01 Da, MS/MS tolerance = ±0.01 Da and peptide charge 2+ and 3+). Search parameters were as follows: enzyme; trypsin; variable modifications: deamidation (NQ), oxidation (M); maximum missed cleavages = 1. A peptide ion score of ≤ 10 as a cut-off as calculated by Mascot was also used to filter. The false discovery rates were determined using the Mascot decoy database option and were ≤1%. Protein identifications were based on a minimum of two unique peptides. Mass spectrometry search results generated by Mascot (*.dat) were imported into ProHits Lite-VM 2.0.3 (http://prohitsms.com/) run via Virtual Box 4.3.6 (https://www.virtualbox.org/) on OSX 10.8.5. A comparison was then performed in Prohits (31) between the lists of proteins for Luzp4 IP and Control IP. Filters used were: Artefacts proteins, Keratin, Albumin and Cytoskeleton. Proteins that passed the filtering and the analysis are presented in Supplementary Table S1. The proteins present in Control IP were then removed from the list of Luzp4 interacting partners. The new filtered list of proteins was used to generate the cytoscape diagram (32) depicted in Figure 2E.

### MS2 mRNA export assays

Assays were carried out as described previously (9).

### Nxf1 remodelling assays and UV-crosslinking experiments

The Nxf1 remodelling assay and UV cross-linking experiments were carried out as described previously (10). The ssDNA used in some assays was 5′-CTCTAGATCAACCACTTTGT-3′. The integrated pixel densities function of the software ImageJ (33) was used to measure and normalize the pixel densities of the phosphorimage signals to the pixel densities of the corresponding Coomassie-stained gel signals. The radiolabelled RNA-crosslinking fold increase was calculated as the increase of crosslinking observed for a given condition relative to the crosslinking observed for the relevant bovine serum albumin (BSA)-containing reactions (RNA or ssDNA).

### Fluorescence in situ hybridisation and immunostaining

Fluorescence in situ hybridisation (FISH) and immunostaining were carried out as described previously (13). Using the ImageJ software (NIH), the nuclear rim of cells in the poly(A)^+^ panels of Figure 4A was determined by creating outline-masks from the corresponding DAPI images which were then applied onto the poly(A)^+^ signal images. The resulting images were used to perform fluorescence intensity scans with a line width of 5 pixels and a length of 56 pixels. To perform the quantifications displayed in Figure 4B and Supplementary Figure S9B, DAPI images of wide-field pictures (20x Leica objective) were thresholded, binary transformed, and cells were automatically counted using the ImageJ software. Pictures containing at least 200 cells (Figure 4B) or at least 60 cells (Supplementary Figure S9B) were collected and their corresponding poly(A)^+^ signal images were thresholded and used to only count cells displaying saturated poly(A)^+^-RNAs nuclear accumulation. A one-way ANOVA analysis followed by a Fisher's LSD test was used to determine which cell lines were prone to display a poly(A)^+^-RNA export block compared to the Control RNAi cell line.

### Luzp4 expression screening in tumour samples

Origene TissueScan Cancer 96-well plates that contained pre-normalized cDNA samples isolated from 18 different body tissues were used according to the manufacturer's instructions. Supplementary Table S2 provides patient details and tumour grades. The PCR primers used were as follows: Alyref_forward 5′-GCCTGCACAGAGCGTAAACA-3′, Alyref_reverse 5′-CTCGCATTATTATAGGCGTCCAG-3′, Luzp4_forward 5′-CGCCTTCAAGACAGCAATCA-3′, Luzp4_reverse 5′-CACTGCACTTCTCCTGCTCAA-3′, Uif_forward 5′-AGCAGTGCAATGCCCAGTAA-3′ and Uif_reverse 5′-ACCGCTCATTCAACGTCATC-3′. U1_forward 5′-ACCTGGCAGGGGAGA-3′ and U1_reverse 5′-GGGGAAAGCGCGAAC-3′ Expression values plotted in Supplementary Figure S7 are shown as 2^−ΔCT^ using U1 snRNA as the normalizer.

## RESULTS

### Luzp4 associates with Nxf1 and TREX subunits

**Figure 1.**
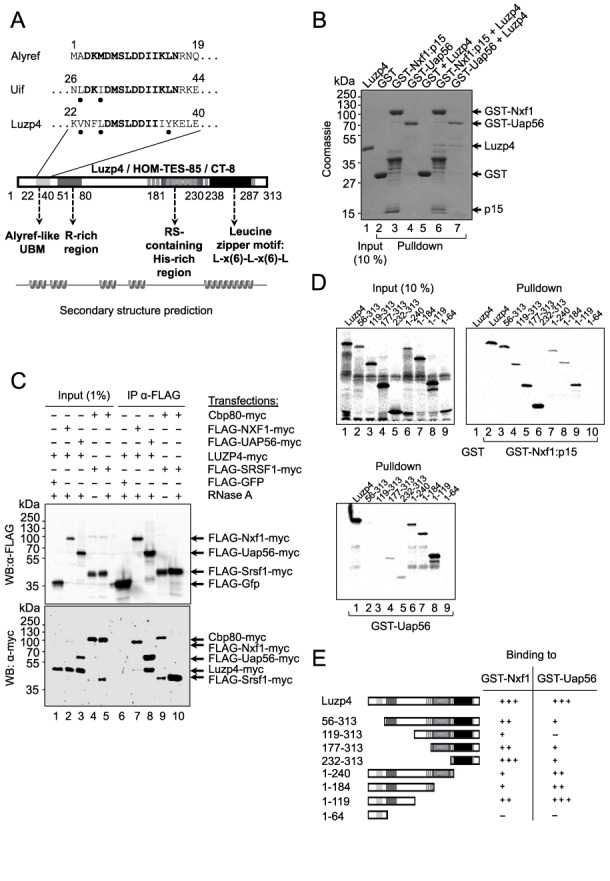
Interaction of Luzp4 with mRNA export factors. (**A**) Schematic of Luzp4 putative domains organisation. The UBM previously identified in Alyref and Uif is aligned with the homologous sequence identified in Luzp4. Residues in bold show strict identity to Alyref sequence. Black dots show biochemically conserved positions. Arginine-Serine (RS) dipeptides are indicated as vertical grey bars. The alternative names used for Luzp4 are also shown. (**B**) GST pulldown assays with the indicated proteins. (**C**) Co-IP assays using the indicated expression vectors. Western blots were probed with the indicated antibodies. (**D**) GST-pulldown analysis of ^35^S labelled Luzp4 truncations as indicated with either GST-Nxf1:p15 (right panel) or GST-Uap56 (lower panel). The ^35^S labelled protein inputs are shown in the left panel. In each case the panel shown is a phosphoimage. Corresponding Coomassie-stained gels are presented in Supplementary Figure S2B. (**E**) Schematic of the truncation constructs used to map binding sites for Nxf1 and Uap56 and summary of the binding data.

A BLAST search with the UBM from the N-terminal domain of Alyref (9) identified Luzp4 (also called HOM-TES-85 or CT-8), a CTA with unknown function. Luzp4 is reported to reside within nuclear speckles, a site enriched in factors involved in pre-mRNA processing and mRNA export (34). Luzp4 orthologues were clearly identifiable in other animals. However, analysis of the X-chromosome in mice revealed a gene expansion at the locus where Luzp4 lies in other species and one Luzp4 paralogue in mice is known as ovary and testis transcribed (Supplementary Figure S1A). Luzp4 harbours a single UBM in its N-terminal domain, a central domain rich in arginines, serine dipeptides and histidines (RS-H) and a C-terminal leucine zipper motif (Figure 1A and Supplementary Figure S1B). We found that following an internal deletion of amino acids (aa) 156–236, which encompasses the RS-H region, GFP-Luzp4 localized to the cytoplasm, whereas wild-type Luzp4 was nuclear, suggesting the RS-H region contains the nuclear localisation sequence of Luzp4 (Supplementary Figure S1C). Interestingly, deletion of the UBM region (aa 22–40) altered the nuclear distribution of GFP-Luzp4, leading to the formation of fewer and larger GFP-Luzp4 foci.

To establish whether Luzp4 directly associated with Uap56 or Nxf1, we used GST pulldown assays with purified Luzp4 protein produced in *E. coli* (Figure 1B). We found that both GST-Uap56 and GST-Nxf1 bound Luzp4 whereas the GST control did not. We also observed the Co-IP of Luzp4 with FLAG-Nxf1-myc and FLAG-Uap56-myc from human cells (Figure 1C). This interaction was not affected by RNase treatment, indicating it was not RNA-dependent, whereas Co-IP of FLAG-Sfrs1 with Cbp80 was RNA dependent (Figure 1C, lanes 9, 10) as reported previously (13). Since the expression of Luzp4 is restricted to testis in normal human tissues (34) where Nxf2 is expressed, we used Co-IP analysis to establish that Luzp4 also binds Nxf2 (Supplementary Figure S2A). In order to map the regions of Luzp4 responsible for interaction with Uap56 and Nxf1, a deletion series was designed and made on the basis of the protein sequence annotation in Figure 1 and Supplementary Figure S1, the secondary structure prediction in Figure 1, and constraints imposed by repetitive DNA motifs within Luzp4 cDNA sequence restricting primers design. These deletion mutants were used in GST-pulldown experiments (Figure 1D, Supplementary Figure S2B). Nxf1 associated strongly with the C-terminal domain of Luzp4 and weakly with the N-terminus, whereas Uap56 associated with aa 1–119 strongly, which encompasses the UBM but only interacted weakly with C-terminal fragments of Luzp4. We conclude that Luzp4 forms direct interactions with both Uap56 and Nxf1 *in vivo* and thus has similarities with the mRNA export adaptors Alyref and Uif.

### Luzp4 binds RNA, associates with TREX subunits and functions with Nxf1 *in vivo*

**Figure 2.**
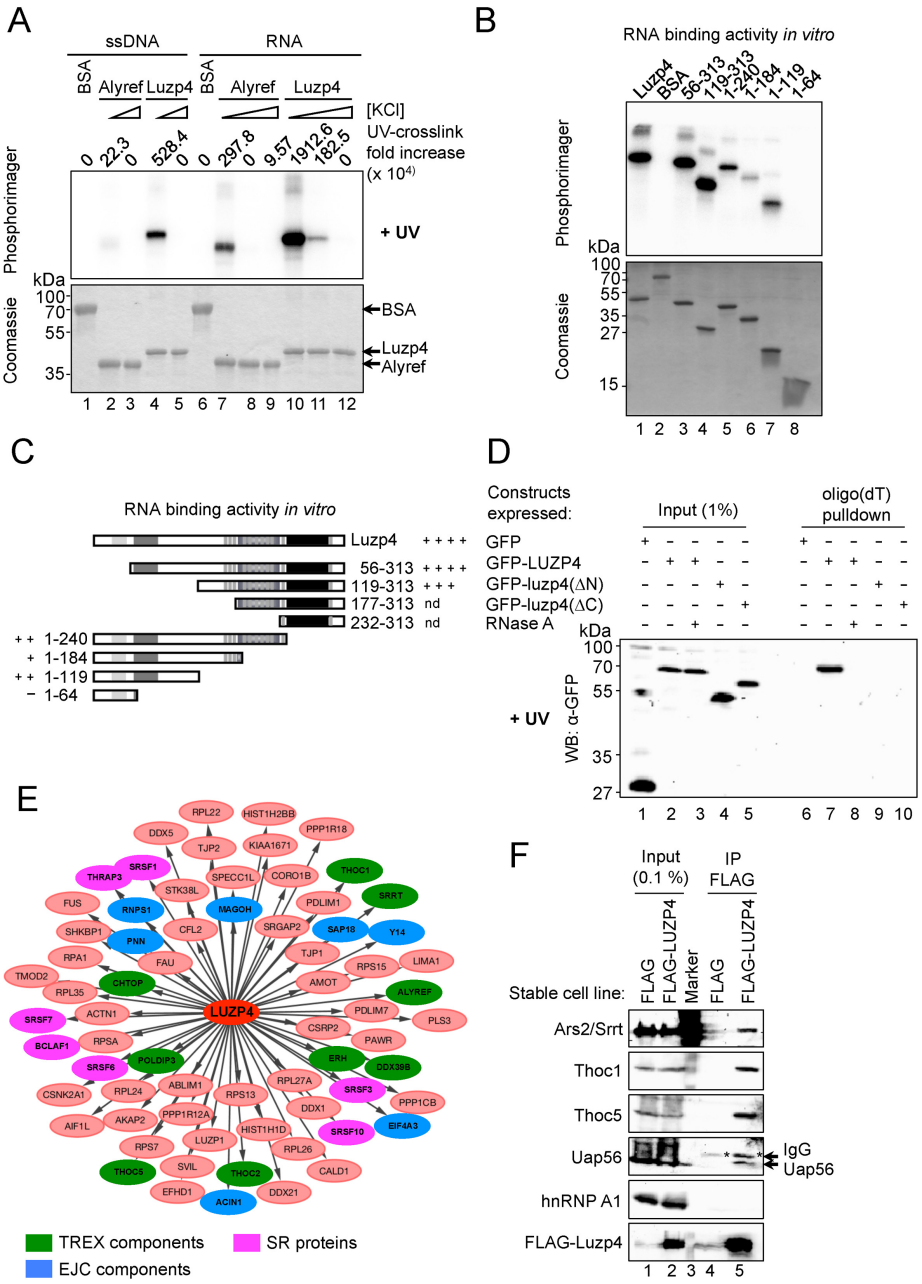
Luzp4 is an RNA binding protein that associates with TREX subunits. (**A**) UV-crosslinking assays using recombinant Alyref, Luzp4 or BSA control and ^32^P-labelled single-stranded RNA or DNA oligonucleotides. Lanes 2, 4, 7, 10 had 100 mM KCl, lanes 8, 11 had 300 mM KCl and lanes 3, 5, 9, 12 had 500 mM KCl in the reactions. The upper panel shows the phosphoimage of the same gel which is Coomassie stained in the lower panel. The quantification shows the increase of crosslinking relative to the relevant BSA-containing reaction (ssDNA or ssRNA). Results where UV crosslinking was omitted were exposed and acquired at the same time and are presented in Supplementary Figure S3A. (**B**) UV crosslinking of ^32^P-labelled RNA oligonucleotide with the indicated purified truncations of Luzp4 in 100 mM NaCl. The upper panels show the phosphoimage of the Coomassie-stained gels shown in the lower panels. (**C**) Schematic of the Luzp4 truncations used to map the RNA binding activity and summary of the RNA binding activity for truncations measured using UV crosslinking. nd: not determined because the constructs are insoluble and precipitated repeatedly during refolding step following purification. (**D**) Luzp4 binds poly(A)^+^-RNAs *in vivo*. The indicated GFP fusions of Luzp4 were analysed for binding to poly(A)^+^-RNAs *in vivo* using UV crosslinking. Proteins were visualized by western blot with anti-GFP antibody. Results where UV crosslinking was omitted were exposed and acquired at the same time and are presented in Supplementary Figure S3B. (**E**) Summary of the interacting proteins identified using mass spectrometry following IP of FLAG-Luzp4 stably expressed in Flp-In™ T-REx™ 293 cells (see Materials and Methods). (**F**) Validation of the mass spectrometry samples. Proteins co-immunoprecipitating with FLAG or FLAG-Luzp4 were detected using western blotting with antibodies to the indicated proteins and FLAG-Luzp4 was detected using FLAG antibody.

Because of its cellular localisation and its ability to bind Nxf1 and Uap56, we thought that Luzp4 could be a new RNA-binding protein, despite a lack of obvious RNA-binding features in its protein sequence. To investigate whether Luzp4 binds RNA, we used UV-crosslinking assays and compared its ability to bind both single-stranded DNA and RNA with that for Alyref. Luzp4 crosslinked strongly with single-stranded RNA at low KCl concentrations and this crosslinking was dependent on UV irradiation (Figure 2A, Supplementary Figure S3A). However, the crosslinking was inhibited by higher KCl concentrations suggesting an ionic interaction between Luzp4 and RNA. Alyref also showed inhibition of RNA binding at higher KCl concentrations suggesting an ionic interaction which is consistent with the earlier observation that arginines are involved in the Alyref:RNA interaction (35). Alyref showed very weak binding to single-stranded DNA, whereas Luzp4 showed stronger binding which was also sensitive to increasing KCl. However the single-stranded DNA binding activity of Luzp4 is only 28% of the RNA binding activity, indicating Luzp4 has clear preference for RNA over single-stranded DNA binding in this assay. We mapped the domain responsible for interaction with RNA using truncations of Luzp4 (Figure 2B and C) and found the major RNA binding activity resided between aa 119–313 with a weaker RNA binding activity between aa 1–119. We confirmed that GFP-Luzp4 associates with the mRNP *in vivo* using an mRNP capture assay (12) (Figure 2D, Supplementary Figure S3B). We also observed that loss of the domains responsible for either Uap56 or Nxf1 interactions (aa1–119 or 241–313, respectively) prevented GFP-Luzp4 from associating with the mRNP. Deletion of the Uap56 and Nxf1 binding domains also altered the localisation of GFP-Luzp4 within the nucleus from a punctate to a more diffuse localisation (Supplementary Figure S3C).

To further characterize Luzp4, we immunoprecipitated FLAG-Luzp4 stably expressed from a Flp-In™ T-REx™ 293 cell line and screened for binding partners by mass spectrometry. This analysis identified numerous TREX components, together with other proteins involved in RNA metabolism including components of the exon junction complex and several SR proteins (Figure 2E, Supplementary Table S1). Interestingly, Ars2/Srrt was present in the Luzp4 IP and has previously been shown to Co-IP with TREX (3). Some of these interacting proteins were validated further by western blot performed on the samples used for the mass spectrometry analysis (Figure 2F). Overall, the Luzp4 interactome obtained here is consistent with its localisation in the nuclear speckles where splicing occurs (34,36) and is also consistent with a role in gene expression and mRNA processing. These results further suggest that Luzp4 assembles with TREX *in vivo*, like Alyref and Uif (9).

**Figure 3.**
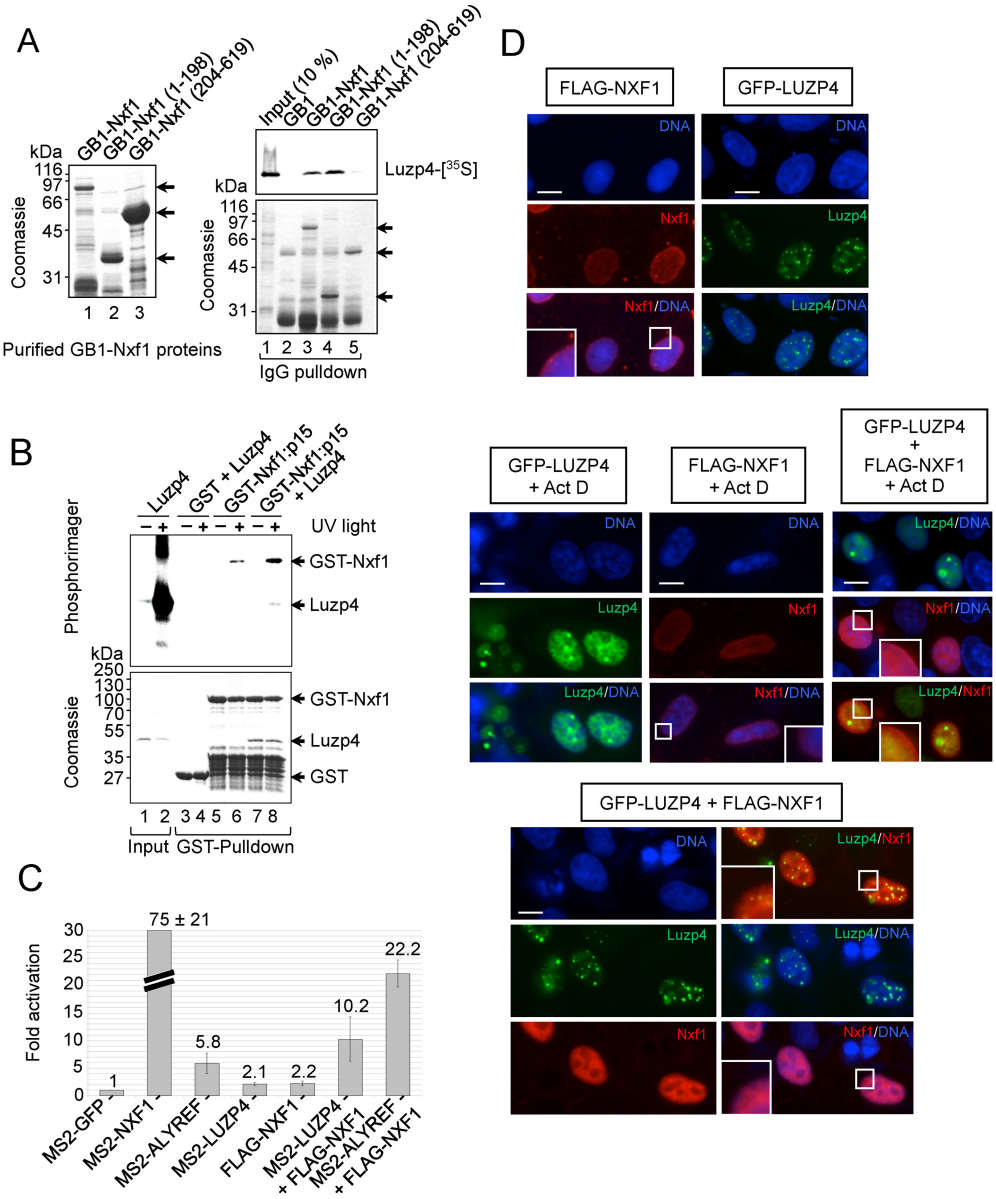
Luzp4 is an mRNA export adaptor. (**A**) The purified GB1-tagged Nxf1 proteins used in pulldown assays are shown (left panel). IgG-Sepharose pulldown assays using ^35^S-Luzp4 and the indicated GB1-tagged Nxf1 proteins (right panels). The upper panel is the phosphoimage of the same gel which is Coomassie stained in the lower panel. (**B**) Nxf1 remodelling assays. Luzp4 pre-incubated with a ^32^P-labelled RNA oligonucleotide was incubated with immobilized GST or GST-Nxf1:p15. The indicated complexes were then purified by GST-pulldown, eluted, UV-crosslinked and analysed by sodium dodecyl sulphate-polyacrylamide gel electrophoresis. The upper panel is the phosphoimage of the same gel shown Coomassie stained in the lower panel. Input was 1%. (**C**) Tethered mRNA export assays (also see Supplementary Figure S4A). The indicated MS2 fusion protein expression vectors were co-transfected with the MS2 reporter pLUCSALRRE6MS2, normally retained in the nucleus, together with a β-galactosidase expression vector. Luciferase activities were measured from the MS2 reporter and normalized for transfection efficiency with the β-galactosidase activities. The graph shows fold activation relative to the levels seen with MS2-GFP and values represent averages from experiments carried out in triplicate on five separate occasions. (**D**) Immunofluorescence images of HeLa cells transfected with the indicated expression vectors. FLAG-Nxf1 was detected using FLAG antibody. When indicated, cells were treated with 5 μg/ml of actinomycin D for 3 h. Certain regions of images indicated by a white box are shown at higher magnification in the bottom left of a panel to observe the presence or reduction of FLAG-Nxf1 at the nuclear rim.

mRNA export adaptors such as Alyref and Uif preferentially bind to aa 1–198 of Nxf1 whereas co-adaptor proteins such as Chtop or Thoc5 mainly bind Nxf1 via the central NTF2L domain. Pulldown assays established that Luzp4 associates with aa 1–198 of Nxf1 (Figure 3A). On binding aa 1–198 of Nxf1, adaptors such as Alyref hand their bound mRNA over to Nxf1. Alyref binding also stimulates partial exposure of the Nxf1 RNA binding domain which leads to enhanced RNA binding by Nxf1 (12,13). We assessed the RNA binding activity of a GST-Nxf1-p15:Luzp4 complex using UV cross-linking (Figure 3B) and found that on binding Luzp4, the RNA crosslinking activity of Nxf1 increased (Figure 3B, compare lanes 6 and 8). Furthermore, when Luzp4 is complexed with Nxf1, it shows significantly reduced RNA cross-linking activity (Figure 3B, compare lanes 2 and 8). We conclude that upon binding to Nxf1, Luzp4, in common with other mRNA export adaptors, has the ability to enhance the RNA binding activity of Nxf1 while simultaneously handing the RNA over to it.

To explore the functional relationship between Luzp4 and Nxf1 *in vivo*, we utilized a tethered mRNA export assay in which an mRNA export factor can overcome nuclear retention of an inefficiently spliced pre-mRNA leading to expression of luciferase retained within an intron (Figure 3C, Supplementary Figure S4) (3,14). As reported previously (12), MS2-Nxf1 stimulated luciferase expression strongly (∼75-fold), whereas MS2-Alyref stimulated expression by 5.8-fold, reflecting its requirement to subsequently recruit Nxf1 prior to mRNA export (Figure 3C). MS2-Luzp4 showed a modest but reproducible ∼2-fold increase in reporter expression. Overexpression of untethered FLAG-Nxf1 also led to a ∼2-fold increase in luciferase expression and this effect has been reported previously with a similar reporter (37). Strikingly, when either Luzp4 or Alyref were tethered to the reporter mRNA via MS2, and FLAG-Nxf1 was simultaneously overexpressed, there was a large increase in luciferase expression (10.2- and 22.2-fold activation, respectively) indicating that Luzp4 or Alyref can work synergistically with Nxf1 in this assay. The strong synergistic effect observed on overexpression of Nxf1 indicates that endogenous Nxf1 levels may be limiting in this assay. This could in part be due to inefficient recruitment of Nxf1 to a tethered export adaptor which may fail to associate with additional TREX subunits such as Thoc1, Chtop and Thoc5 which also mediate Nxf1 interactions (13,38). Increasing the concentration of Nxf1 may compensate for the loss of interactions with additional TREX subunits with the isolated tethered export adaptor, leading to enhanced mRNA export.

We investigated the impact of Luzp4 expression on Nxf1 localisation *in vivo* (Figure 3D). Transiently transfected FLAG-Nxf1 showed a diffuse nuclear staining and staining at the nuclear rim as reported previously (39) whereas GFP-Luzp4 showed a punctate staining pattern, as observed previously (34). When GFP-Luzp4 and Flag-Nxf1 were co-expressed, we observed loss of the nuclear rim staining for Nxf1. These data are consistent with the earlier observation that overexpression of Srsf1, which also binds and functions with Nxf1, can prevent its association with the nuclear rim (40). Moreover, whilst inhibition of transcription by actinomycin D had no effect on FLAG-Nxf1 localisation, it resulted in a diffuse nuclear distribution for GFP-Luzp4. Interestingly, this change in GFP-Luzp4 localisation upon transcription inhibition was concomitant with a persistence of FLAG-Nxf1 staining at the nuclear rim of cells co-expressing both proteins. We also examined GFP-Luzp4 and Nxf1 localisation in cells at different stages of the cell cycle and found that GFP-Luzp4 relocalizes to the cytoplasm in newly divided cells, whereas Nxf1 remains nuclear and retains nuclear rim staining (Supplementary Figure S5A). The leucine zipper domain of Luzp4 is involved in this phenomenon since the mutant Luzp4ΔC(aa 1–240) is almost exclusively nuclear in newly divided cells (Supplementary Figure S5B). Together, these data indicate that there is a functional relationship between Nxf1 and Luzp4 *in vivo* and Luzp4 can alter the steady-state localisation of Nxf1 in a transcription-dependent manner.

### Luzp4 complements loss of Alyref *in vivo*

**Figure 4.**
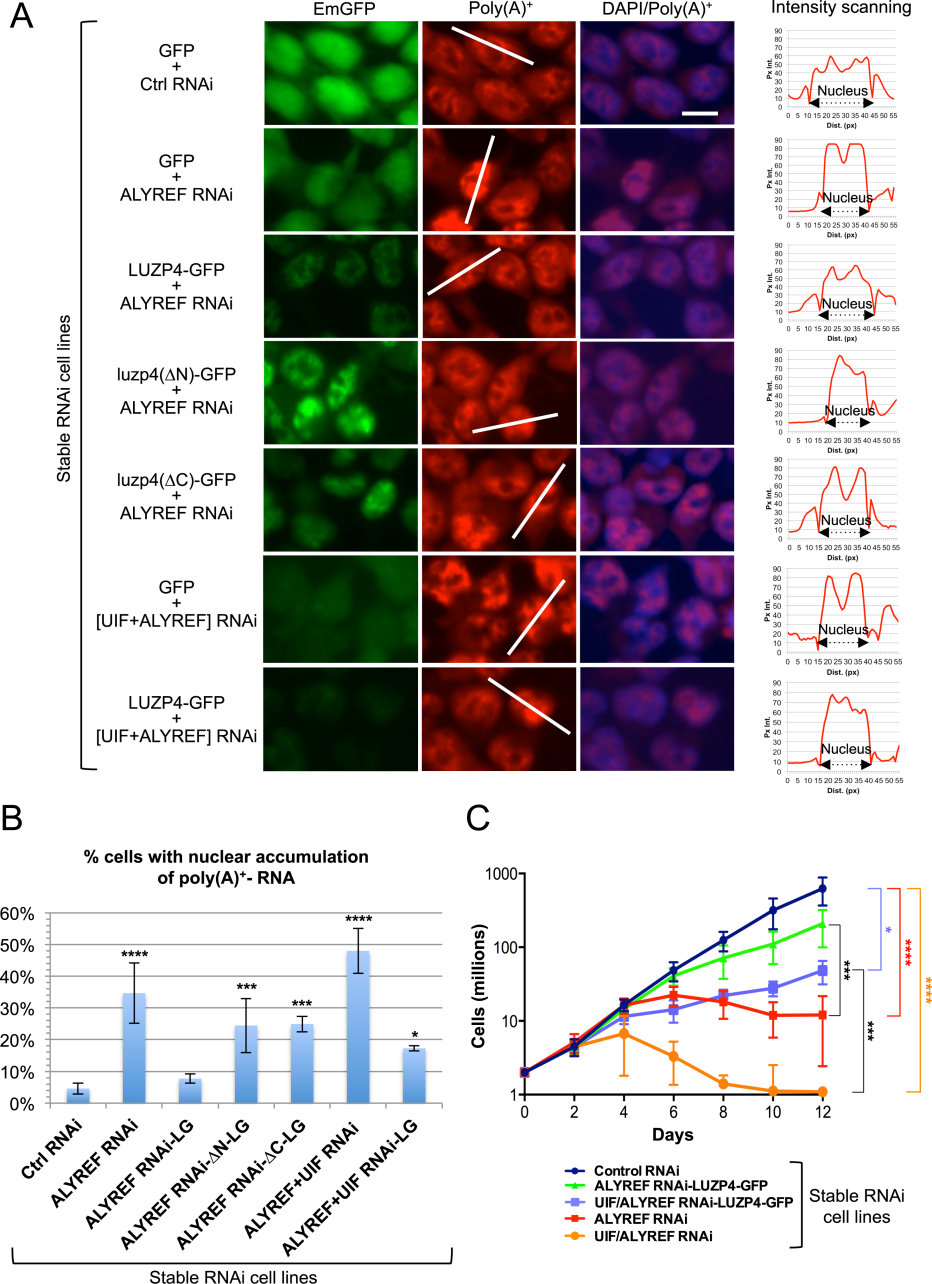
Luzp4 can complement knockdown of other mRNA export adaptors. (**A**) The indicated stable inducible RNAi cell lines were analysed for GFP fusion expression (left panels), for poly (A)^+^-RNA using FISH analysis with oligo-(dT)_50_ (central panels), and an overlay of DAPI stained cells and the poly(A)^+^ signal is shown in the right panels. RNAi-mediated knockdowns were induced for 96 h. Fluorescence intensity scanning of indicated cells (white bars) are shown on the right. The y-axis of the graphs represents the pixel intensity (Px Int.) and the x-axis the distance in pixels (Dist. (px)). All equivalent panels are shown at the same exposure. (**B**) Quantification for the results presented in panel A. Three images (with at least 200 cells each) per condition were used to count the proportion of cells displaying a nuclear accumulation of poly(A)^+^ RNAs. Mean values ± standard deviation are shown. P_Ctrl RNAi versus ALYREF RNAi-LG_ = 0.5262. (**C**) Growth of stable cell lines following induction of the indicated miRNAs and complementing cDNAs with tetracycline. Mean values ± standard deviation from five independent experiments are shown. The statistical differences in growth rates are shown on the right. P_Control RNAi versus ALYREF RNAi-LUZP4-GFP_ = 0.080. *P< 0.05, ***P < 0.001, ****P < 0.0001. Supplementary Figure S6B provides a schematic of the complementation system.

We noted that Alyref RNAi did not lead to up-regulation of endogenous Luzp4 expression in 293 cells (Supplementary Figure S6A). This provided an opportunity to artificially express Luzp4 and test whether it would complement the mRNA export and growth defects observed following Alyref RNAi in a 293 cell. To do this, we generated stable Flp-In™ T-REx™ 293 cell lines in which Luzp4 was expressed as a GFP fusion in a transcript with miRNAs in its 3′ UTR targeting either Alyref or Alyref+Uif (Supplementary Figure S6B). We analysed these cell lines for mRNA export defects using oligo(dT) FISH (Figure 4A and B). Alyref RNAi led to an mRNA export block as reported previously with clear nuclear accumulation of poly(A)^+^-RNA (9). However, Luzp4 expression complemented this mRNA export defect, indicating that Luzp4 can function as an mRNA export factor in place of Alyref. In contrast, a GFP fusion of Luzp4 missing aa 1–118 (Luzp4(ΔN)-GFP) which does not bind Uap56 (Figure 1D and E), or a GFP fusion of Luzp4 aa 1–240 (Luzp4(ΔC)-GFP), which binds Nxf1 and RNA poorly, did not complement the mRNA export defect triggered by Alyref RNAi (Figure 4A and B). Following Alyref + Uif combined RNAi, cells exhibit an extreme mRNA export block with robust nuclear accumulation of large areas of poly(A)^+^ material in the majority of cells ((9) and Figure 4A and B). This mRNA export block was partially complemented by expression of GFP-Luzp4, with fewer cells showing strong nuclear accumulation of poly(A)^+^-RNA. We also examined the expression of the GFP fusions in the stable cell lines (Figure 4A, left panels) and found that the levels of GFP protein were reduced following Alyref RNAi and further reduced following Alyref + Uif RNAi. GFP-Luzp4 also showed reduced expression following Alyref+Uif RNAi compared with just Alyref RNAi. This probably arises from reduced GFP or GFP-Luzp4 mRNA export in cell lines following Alyref and Uif RNAi. Despite this, the lower levels of GFP-Luzp4 following Alyref + Uif RNAi were still able to partially complement mRNA export (Figure 4A and B). We further examined the ability of Luzp4 to complement the growth defect observed following Alyref and Uif RNAi using the stable cell lines generated for the FISH analysis. Whereas Alyref and Alyref+Uif combined RNAi cell lines showed a clear growth defect (Figure 4C), both cell lines showed significantly better growth when they expressed GFP-Luzp4. However, the cell growth was not completely restored to normal levels, particularly when both Alyref and Uif were knocked down by RNAi.

**Figure 5.**
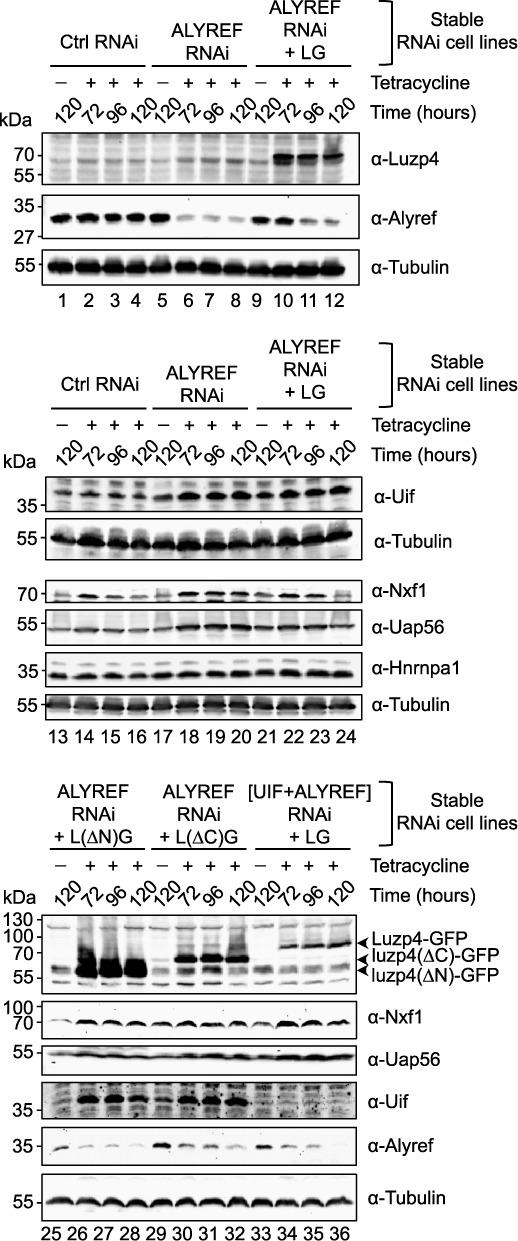
Luzp4 rescues the mRNA export pathway impaired in ALYREF RNAi cells. Western blot analysis of stable cell lines following induction of the indicated miRNAs and complementing cDNAs at the indicated time points. The antibodies used to detect the proteins are indicated on the right-hand side of each panel. Tetracycline is used to induce expression of the miRNA/complementing cDNA. Supplementary Figure S6B provides a schematic of the complementation system. LG = Luzp4-GFP.

When mRNA export is blocked, cells respond by up-regulating a variety of mRNA export factors (9,11). We therefore also assessed what happens in cells following Alyref or Alyref+Uif RNAi and the impact that expression of GFP-Luzp4 had in such cells using the complementation cell lines described above. Following Alyref RNAi we saw a clear up-regulation of Uif, Nxf1 and Uap56 protein levels (Figure 5, lanes 18–20). In contrast, an mRNP binding factor not directly associated with mRNA export, Hnrnpa1, was not affected (Figure 5, lanes 18–20). When GFP-Luzp4 was co-expressed in the Alyref RNAi cell line, the up-regulation of Nxf1 and Uap56 was suppressed, particularly at later time points of expression (Figure 5, compare lanes 20 and 24). However, Uif levels remained raised following Alyref RNAi and were not suppressed by GFP-Luzp4 expression. We also examined the ability of complementing cell lines expressing non-functional mutant forms of Luzp4 to suppress the up-regulation of export factors following Alyref RNAi and found that neither mutant forms of Luzp4 could suppress this response (Figure 5, lanes 26–28, lanes 30–32). Finally, we examined the ability of GFP-Luzp4 expression to suppress up-regulation of export factors following Alyref+Uif RNAi and found that it could not (Figure 5, lanes 34–36). Together, these results indicate that Luzp4 functions as an mRNA export factor in human cells that is able to restore mRNA export function in cells compromised by loss of mRNA export adaptors.

### Luzp4 is preferentially expressed in melanomas

**Figure 6.**
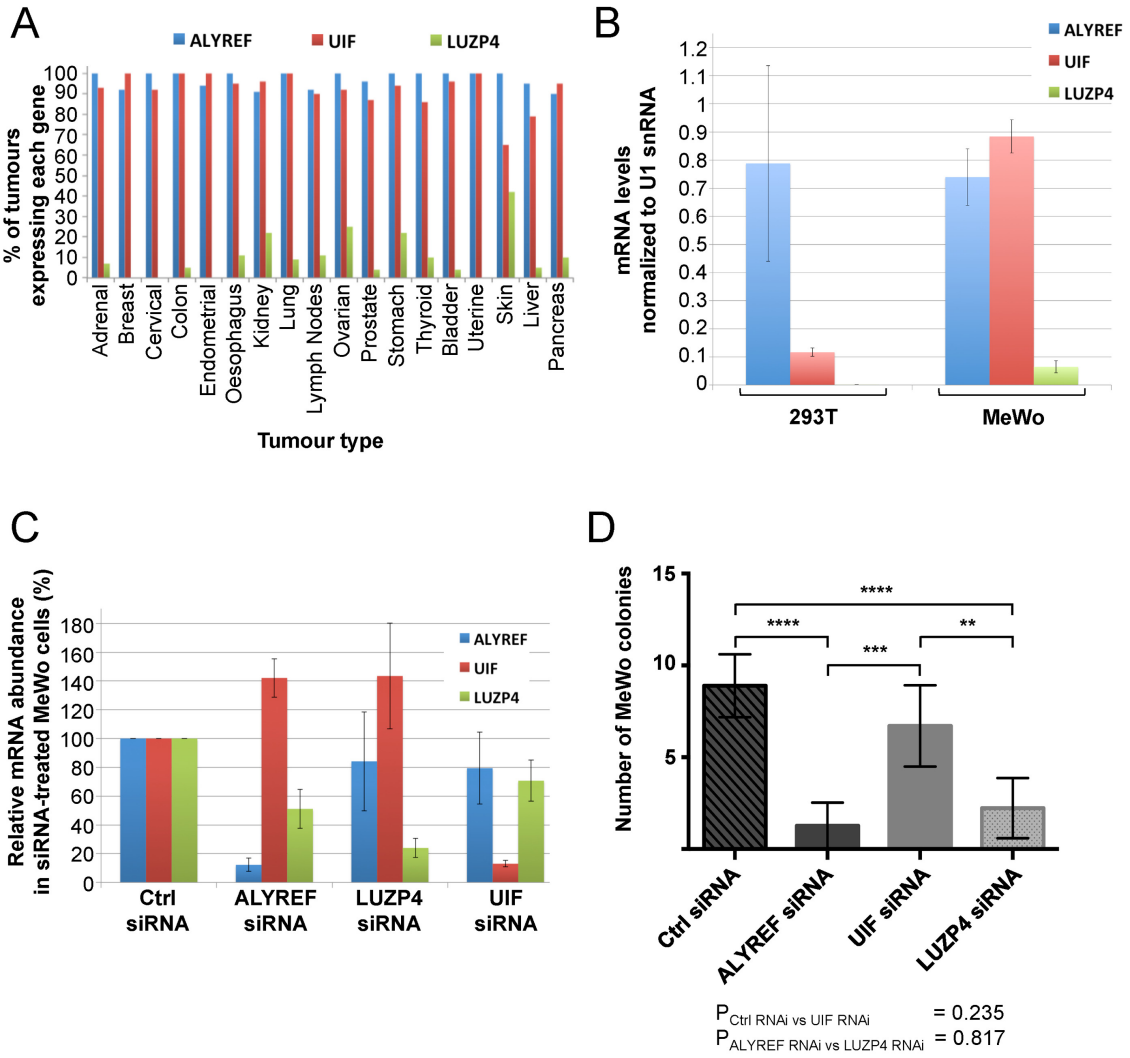
Luzp4 is expressed in cancer cells and involved in MeWo cell growth. (**A**) Summary of the qRT-PCR analysis of Luzp4, Uif and Alyref expression in RNA samples derived from 402 different patients, representing multiple different clinical stages of tumours from many tissues. The full data set for each tissue is shown in Supplementary Figure S7. Genes were defined as expressed when 2^−ΔCT^ >0.04. (**B**) qRT-PCR analysis of expression Alyref, Uif and Luzp4 in MeWo and 293T cells. Values for LUZP4 levels in 293T and MeWo were 0.00055 and 0.06433, respectively. The results represent the average of three independent experiments. The error bars represent the standard deviation. (**C**) qRT-PCR analysis to confirm efficient knockdown of ALYREF, LUZP4 and UIF mRNA in MeWo cells using siRNAs. The values shown are normalized to U1 snRNA abundance. The error bars represent the standard deviation from three independent experiments. (**D**) Colony formation assay for MeWo cells following knockdown of the indicated mRNAs using siRNAs. The values shown for all graphs represent the average of five independent experiments, each containing six replicates per condition and the error bars represent the standard deviation. **P < 0.01, ***P < 0.001, ****P < 0.0001.

Since Luzp4 was originally described as a CTA we examined Luzp4, Alyref and Uif expression in tumour biopsies from 402 different patients, representing multiple different clinical stages of tumours from a wide range of tissues (Figure 6A, Supplementary Figure S7 and Supplementary Table S2). Generally, the expression levels for Luzp4 mRNA were low compared with Alyref and Uif, although we observed at least one tumour biopsy that showed increased Luzp4 expression within most tissue types. The striking exception was malignant melanoma where Luzp4 expression was very high for many patients. This is consistent with the earlier qualitative observation of Luzp4 expression in multiple melanoma samples (34). These data suggest that Luzp4 expression is preferentially activated in melanoma.

To identify cells suitable for further studies on Luzp4, we screened various cancer cell lines for expression of Luzp4 and found that the melanoma cell line MeWo had readily detectable expression (Supplementary Figure S8A). We confirmed the expression of Luzp4 in MeWo cells using qRT-PCR and found that the levels were significantly lower than those for both Uif and Alyref and found no detectable expression of Luzp4 in 293T cells (Figure 6B). We also raised two separate antibodies to Luzp4 and screened for Luzp4 protein (Supplementary Figure S8B) in Mewo cells. Whilst the antibody was able to detect Luzp4 when transiently overexpressed we were unable to unambiguously detect the endogenous protein with our antibodies, suggesting that it is present at low levels as indicated by the qRT-PCR analysis.

We investigated whether Luzp4 RNAi led to a detectable block in mRNA export in Mewo cells using oligo d(T) FISH (Supplementary Figure S9) and whilst we could see a clear block of export following Alyref RNAi, no such block was seen following Luzp4 RNAi. Therefore Luzp4 may be responsible for the export of a restricted set of mRNAs. We further analysed the effects of Luzp4, Alyref and Uif RNAi on the growth of Mewo cells using a colony formation assay (Figure 6D). Alyref RNAi led to a significant growth defect in Mewo cells, whereas Uif RNAi did not, suggesting it was not essential for cellular proliferation, as previously observed in 293T cells (9). Luzp4 RNAi led to a significant drop in the number of Mewo cell colonies formed. Importantly, Luzp4 siRNAs did not affect cellular proliferation of 293T cells (Supplementary Figure S8C), where Luzp4 is not expressed, indicating they do not have off-target effects which affect general cellular growth. We conclude that Luzp4 is required for the growth of Mewo cells.

## DISCUSSION

Luzp4 was first defined as a CTA 13 years ago but its molecular function has remained unknown until now. We have shown that Luzp4 is a new RNA binding protein and we have mapped two regions of the protein involved in this activity. One is an arginine-rich region located after the UBM, as is the case with Alyref. The second region involved in RNA binding maps to the leucine zipper motif and interestingly a helical wheel projection of the amino acids within the leucine zipper reveals a positively charged surface which may be required for RNA interactions (34). We have further demonstrated that Luzp4 functions as an mRNA export factor, capable of complementing knockdown of Alyref and partially complementing a double Alyref/Uif knockdown in 293 cells. Luzp4 has a UBM that allows association with Uap56. Given that Uap56 drives TREX assembly (3,10), this interaction probably ensures the incorporation of Luzp4 in the TREX complex. Consistent with this, the UBM is required for proper nuclear distribution of Luzp4 and we observed Co-IP of Luzp4 with TREX subunits. A common property of mRNA export adaptors such as Alyref, Srsf3 and Srsf7 is their ability to bind the N-terminal region of Nxf1 and enhance its RNA binding activity (12). This involves release of the Nxf1 RNA binding domain from its interaction with the NTF2L domain (13). Here, we observe that Luzp4 also has the ability to enhance the RNA binding activity of Nxf1 through an interaction with the Nxf1 N-terminal region. Thus Luzp4 can be classified as a new mRNA export adaptor.

Luzp4 was originally reported to be expressed in human testis (34) and a more extensive survey of its tissue specific expression by the Human Protein Atlas project (41) (http://www.proteinatlas.org/ENSG00000102021-LUZP4/tissue) confirms this restricted expression pattern. Interestingly, Luzp4 is able to interact with Nxf2 which may be the major mRNA export receptor in this tissue. Whilst testes express Luzp4, they also express Alyref (http://www.proteinatlas.org/ENSG00000183684/tissue/testis) and thus it is unlikely that Luzp4 acts as the sole mRNA export adaptor in this tissue. Why the testis has evolved distinct mRNA export factors remains unclear, though it may be related to the meiotic gene expression programme in this tissue.

The ability of Luzp4 to complement the export defect seen following Alyref RNAi implies that it has the ability to associate with a wide variety of mRNAs in a 293 cell. This indicates there is functional redundancy between Alyref and Luzp4 as reported previously for Uif and Alyref (9). Indeed Alyref is clearly playing a major role in mRNA export in Mewo cells, since its knockdown leads to a significant mRNA export block and prevents cellular proliferation. Interestingly in a number of high grade tumours, Alyref expression levels are reported to be diminished (24). Therefore, given the potential functional redundancy between Alyref and Luzp4, it seems plausible that in certain tumours, Luzp4 may function to promote the export of mRNAs which would normally utilize Alyref. Since Luzp4 RNAi in Mewo cells does not lead to a strong accumulation of mRNA when assayed by FISH it is possible that the export of a restricted set of mRNAs is dependent on Luzp4 in these cells.

Luzp4 was originally discovered using the SEREX technique, whereby circulating antibodies from a seminoma patient were used for expression screening of testis cDNA libraries. It was further shown to be expressed in a wide variety of cancers including melanoma, seminoma, ovarian, lung and glioma and its expression could be activated in normal cells by addition of the DNA methylation inhibitor, 5-aza-2′-deoxycytidine (34). Here, we have extended that analysis of the Luzp4 expression profile in cancer cells and found it is expressed in a wide variety of cancer types, but particularly high levels of expression are observed in melanoma cells. Recent work identified Luzp4 as commonly up-regulated in multiple myeloma (MM) cell lines and the bone marrow of MM patients, a cell-type absent from our screen of tumours (42). Furthermore this study showed that Luzp4 knockdown prevented colony formation for an MM cell line and sensitized the cell line to chemotherapeutic reagents (arsenic trioxide and bortezomib). These data are consistent with our observation that the Mewo melanoma cell line also requires Luzp4 for efficient colony formation. Therefore, at least two types of cancer cells share the requirement for Luzp4 for efficient growth and, given its restricted expression profile in non-disease states, it may be an appropriate target for development of drugs or immunotherapies to treat MM or melanoma.

## SUPPLEMENTARY DATA

Supplementary Data are available at NAR Online.

SUPPLEMENTARY DATA
