# Supplementary material for: Luzp4 defines a new mRNA export pathway in cancer cells
Source: Nucleic Acids Res. 2015 Feb 6;43(4):2353–66. doi: 10.1093/nar/gkv070 (PMC4344508; doi:10.1093/nar/gkv070)
Supplement: SUPPLEMENTARY DATA [file supp_43_4_2353__index.html]

Luzp4 defines a new mRNA export pathway in cancer cells — Luzp4 defines a new mRNA export pathway in cancer cells — SUPPLEMENTARY DATA 

# Luzp4 defines a new mRNA export pathway in cancer cells

## SUPPLEMENTARY DATA

**Files in this Data Supplement:**

- SUPPLEMENTARY DATA
- SUPPLEMENTARY DATA
- SUPPLEMENTARY DATA
