# Supplementary material for: Luzp4 defines a new mRNA export pathway in cancer cells
Source: Nucleic Acids Res. 2015 Feb 6;43(4):2353–66. doi: 10.1093/nar/gkv070 (PMC4344508; doi:10.1093/nar/gkv070)
Supplement: SUPPLEMENTARY DATA [file supp_gkv070_nar-03290-a-2014-File010.pdf]

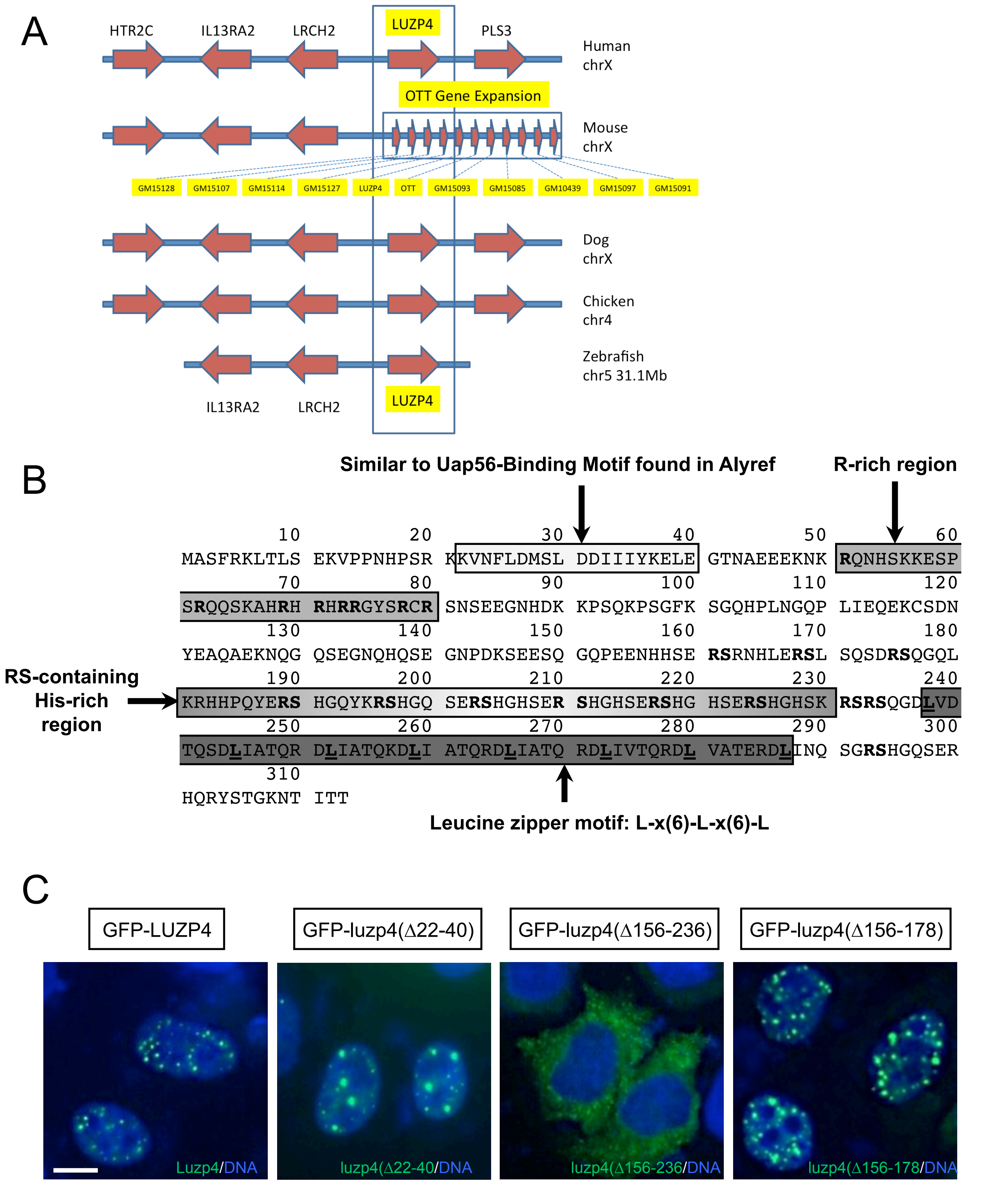

Supplementary Figure S1. Bioinformatic analysis on Luzp4 and mapping of Luzp4 NLS. (A) Syntenic analysis of LUZP4 in vertebrates. LUZP4 genes are expanded in the mouse genome and include the paralogue OTT. **(B)** Amino acid sequence of Luzp4 with key motifs highlighted. **(C)** The UBM-like motif (aa 22-40) is important for Luzp4 nuclear distribution and the NLS of Luzp4 lies within the RS-containing His-rich (RS-H) region. Fluorescence images of the indicated GFP fusions expressed in HeLa cells. The scale bar corresponds to 10 μm.

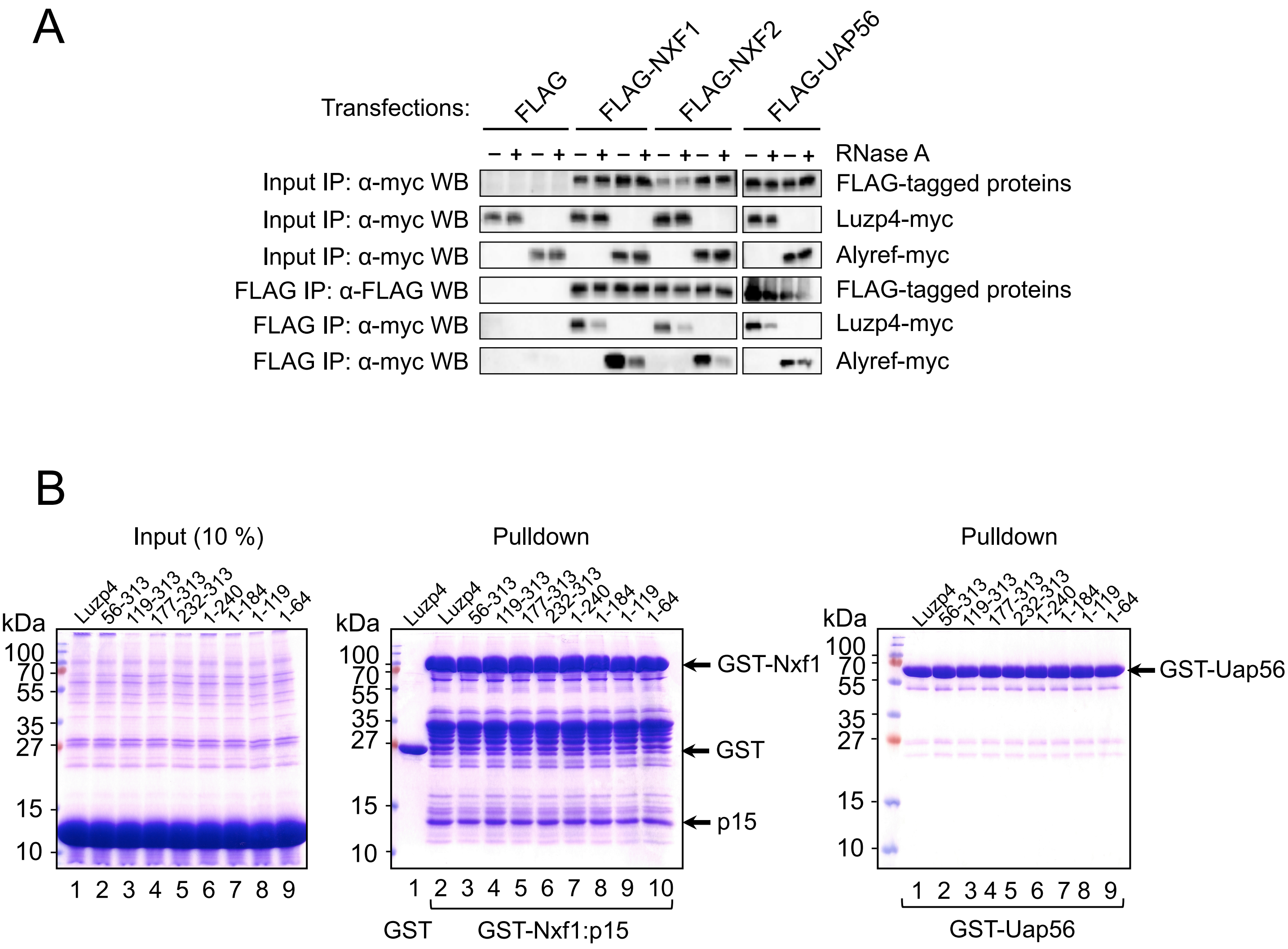

Supplementary Figure S2. Interaction of Luzp4 with other mRNA export factors.

(A) Co-IP analysis of the indicated FLAG tagged cDNAs with Myc tagged Luzp4 and Alyref. Anti-FLAG antibody was used for the Co-IP and Western blots were probed with the antibodies indicated on the left hand side. Inputs were 7.5 %.

(B) Coomassie gels of the GST-pulldowns presented in Figure 1D. GST-pulldown analysis of ^{35}S labelled Luzp4 truncations as indicated with either GST-Nxf1:p15 (central panel) or GST-Uap56 (right panel). The ^{35}S labelled protein inputs are shown in the left panel.

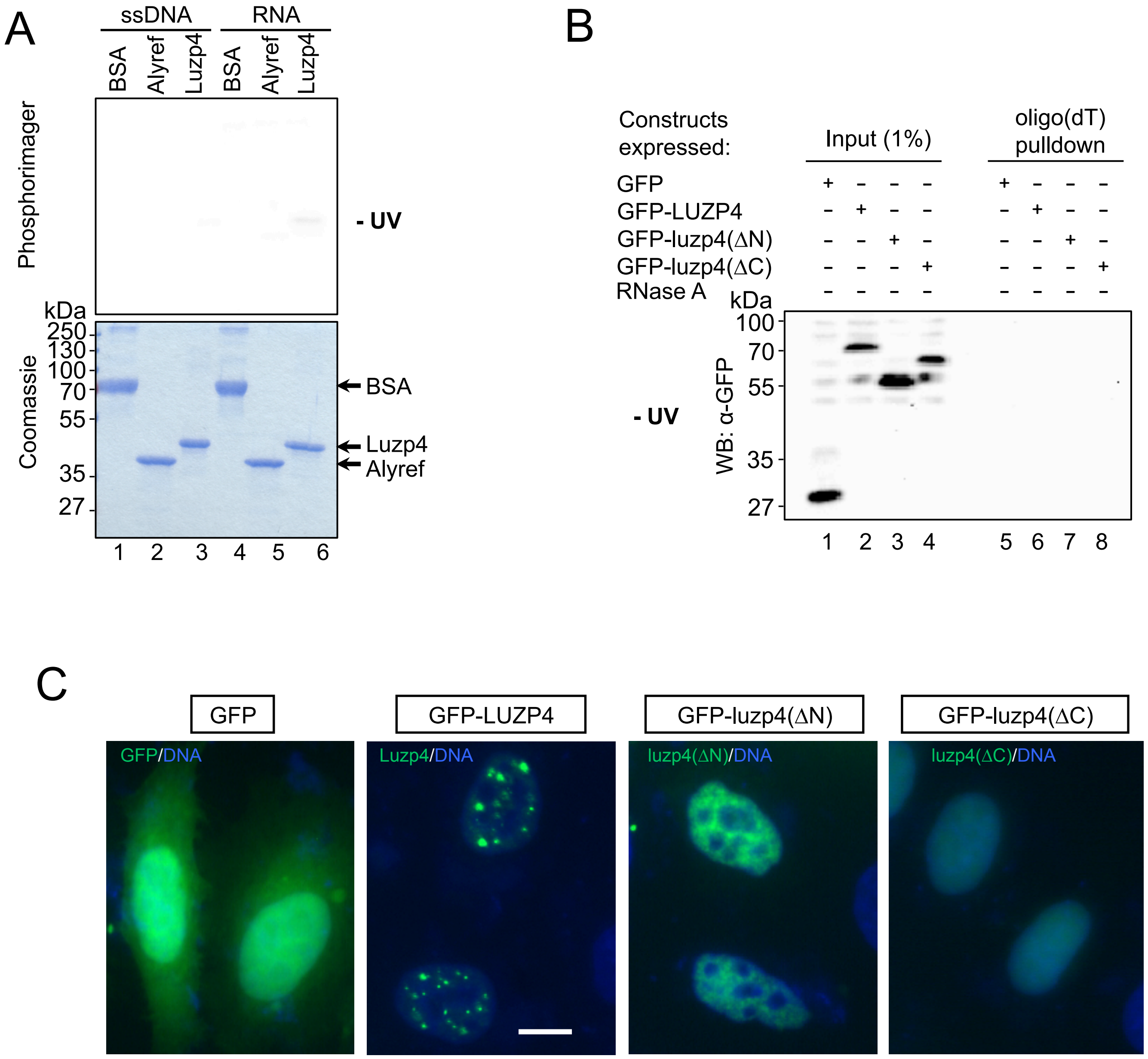

Supplementary Figure S3. RNA binding activity of Luzp4.

(A) Panels relevant to Figure 2A. *In vitro* RNA binding experiment where UV irradiation was omitted. The indicated phosphorimage was acquired at the same time as in Figure 2A.

(B) mRNP capture assays of Luzp4 mutants. Panel relevant to Figure 2D. UV irradiation was here omitted. Proteins were visualised by Western blot with anti-GFP antibody. The image was acquired at the same time as Figure 2D.

(C) Subcellular localisation of GFP-Luzp4 and truncations transiently expressed in Hela cells. The scale bar corresponds to 10 μm.

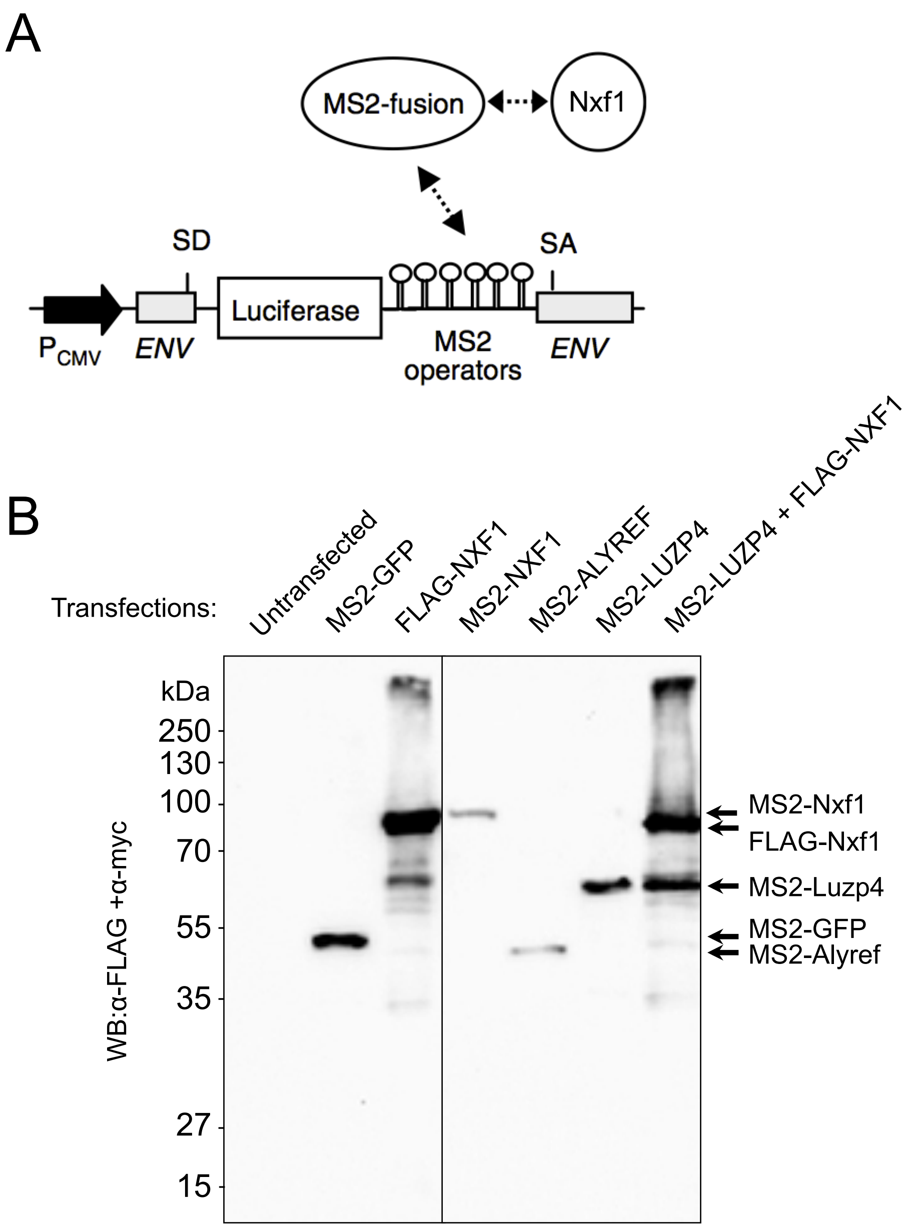

Supplementary Figure S4. Tethered mRNA export assay.
(A) Schematic of the tethered mRNA export assay. The luciferase reporter gene is sited within an inefficiently spliced intron derived from HIV-1. When an mRNA export factor is tethered to the reporter mRNA it overcomes nuclear retention and the exported pre-mRNA leads to luciferase production.
(B) Western blot analysis was performed on 5 µg of protein extracts to confirm expression of the MS2 fusions used in Figure 3C. The membrane was probed with anti-FLAG (Sigma®) and anti-myc (Roche®) antibodies.

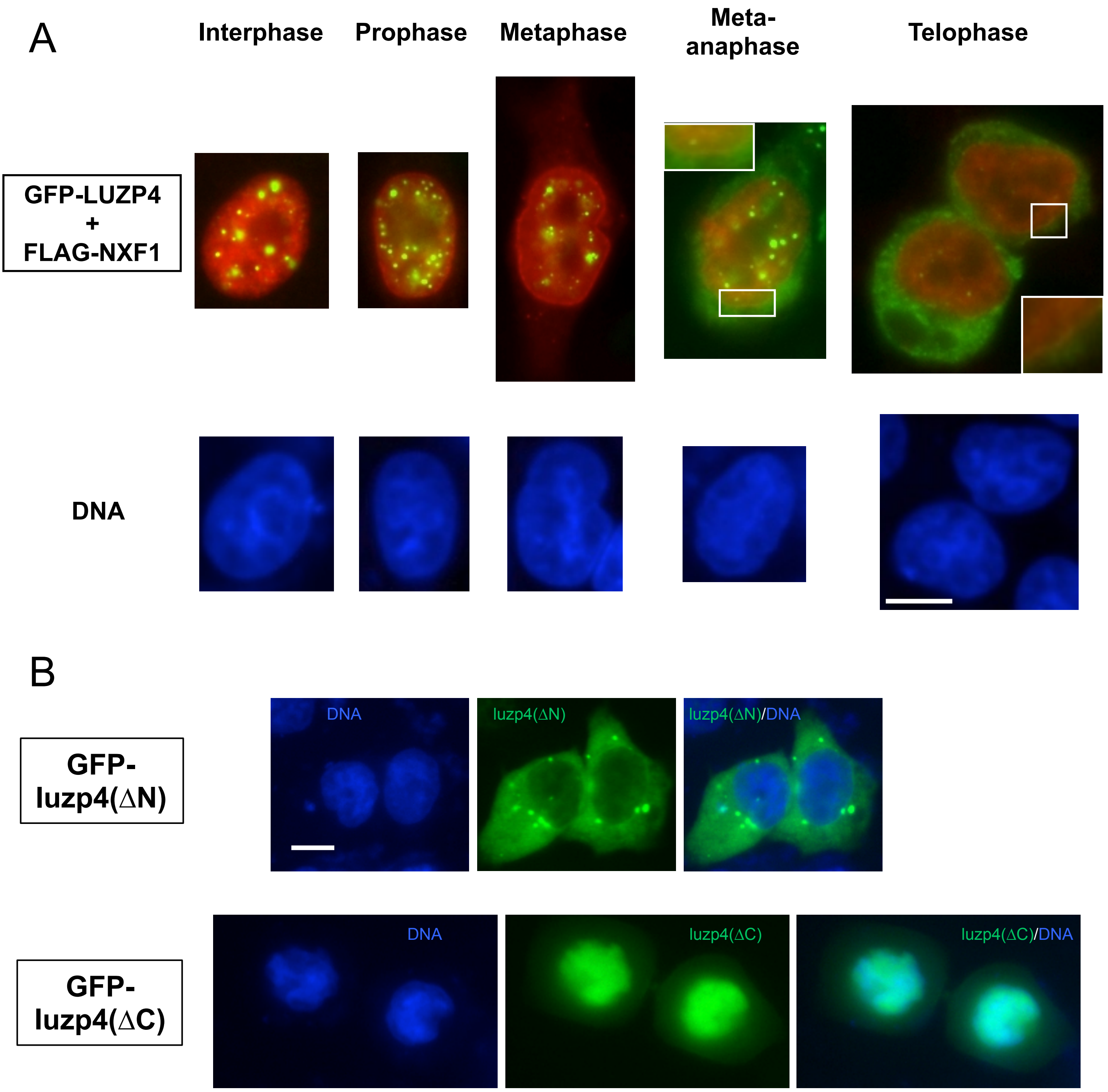

Supplementary Figure S5. Luzp4 relocalises to the cytoplasm in newly divided cells.

(A) During cell division, Luzp4 is relocalised to the cytoplasm and is unable to trigger a depletion of Nxf1's staining from the nuclear rim. GFP-LUZP4 and FLAG-NXF1 were transiently expressed in HeLa cells **(B)** This relocalisation phenomenon requires the leucine-zipper domain of Luzp4. In its absence, the majority of luzp4(ΔC) mutant proteins stays in the nucleus. The indicated GFP fusions were transiently transfected in HeLa cells. The scale bars correspond to 10 µm.

A

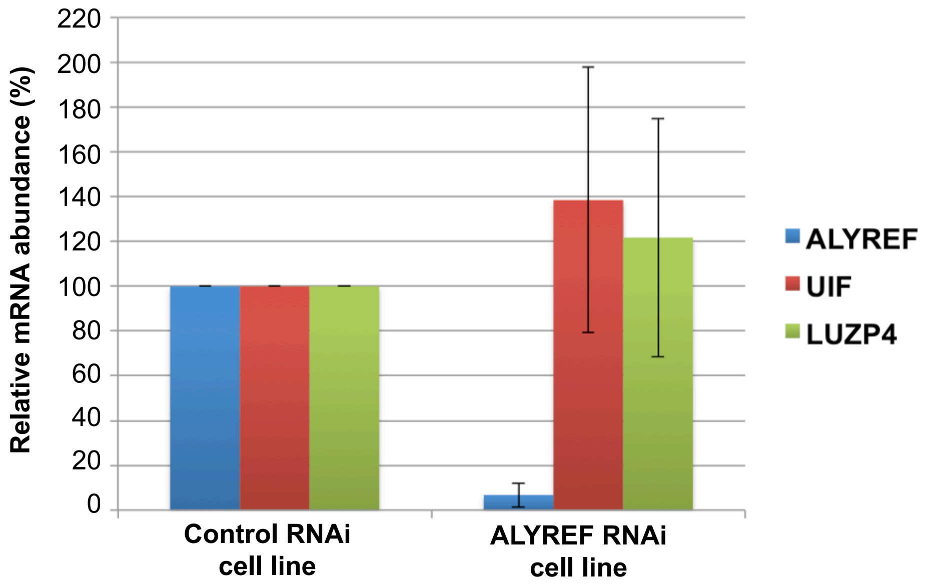

B

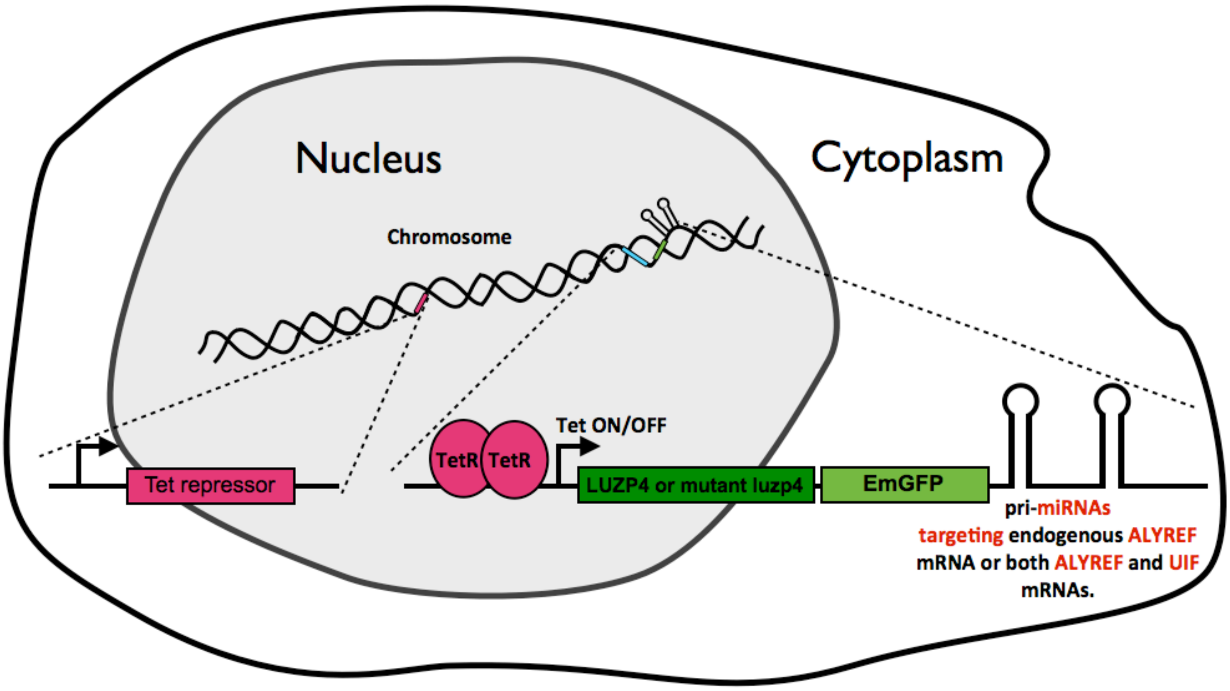

Supplementary Figure S6. Luzp4 complementation system.

(A) qRT-PCR analysis on the mRNA levels for the indicated genes in Flp-In™ T-REx™ 293 Control RNAi or Alyref RNAi stable cell lines.

(B) Schematic of the complementation system used to knockdown endogenous mRNA export adaptors by RNAi and to simultaneously express wild-type or mutant forms of Luzp4 in a Flp-In™ T-REx™ 293 cellular background.

Supplementary data 7. Relative expression of ALYREF, UIF and LUZP4 in cancerous tissues.

qRT-PCR was used to evaluate the abundance of ALYREF, UIF, and LUZP4 at the RNA level using commercially purchased cDNA array plates from *Origene.* The graphs show delta CT values for each gene normalised to the U1 snRNA content for each cDNA taken from cancer tissues from a catalogue of patients. Wells can be cross-referenced to the Origene datasheet (Supplementary Table S2), 'P' stands for 'plate' and the following letter gives the well number.

Adrenal

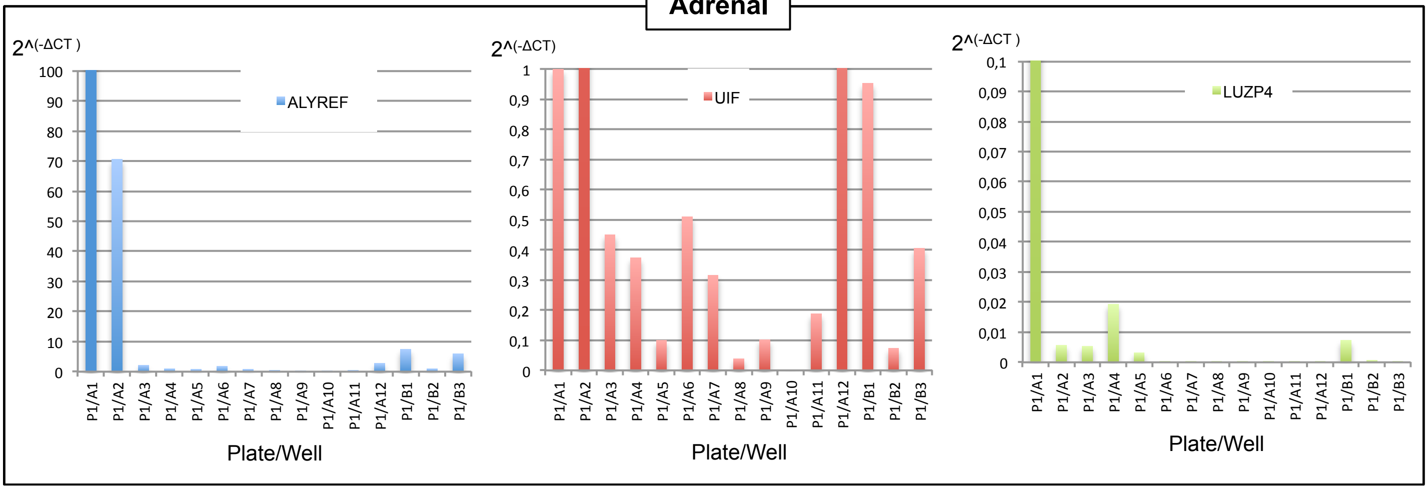

Liver

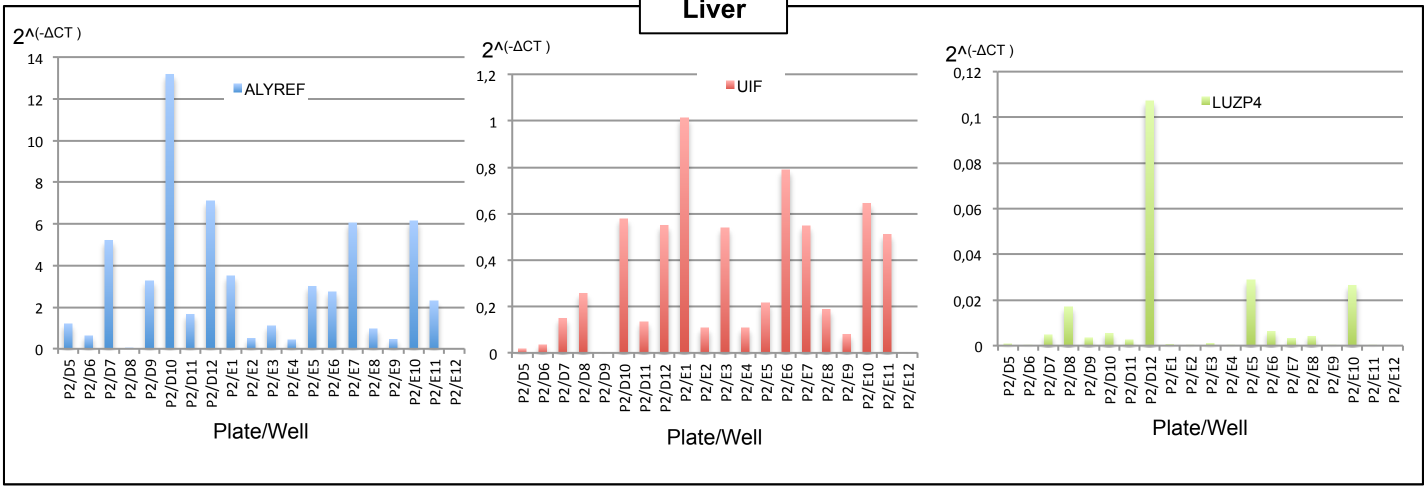

Pancreas

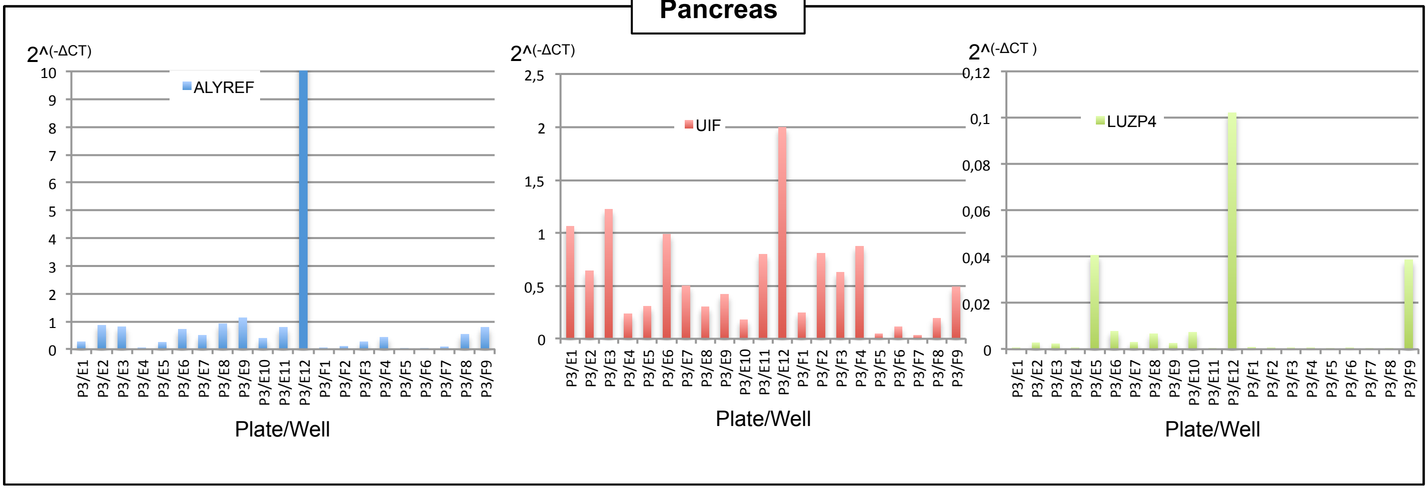

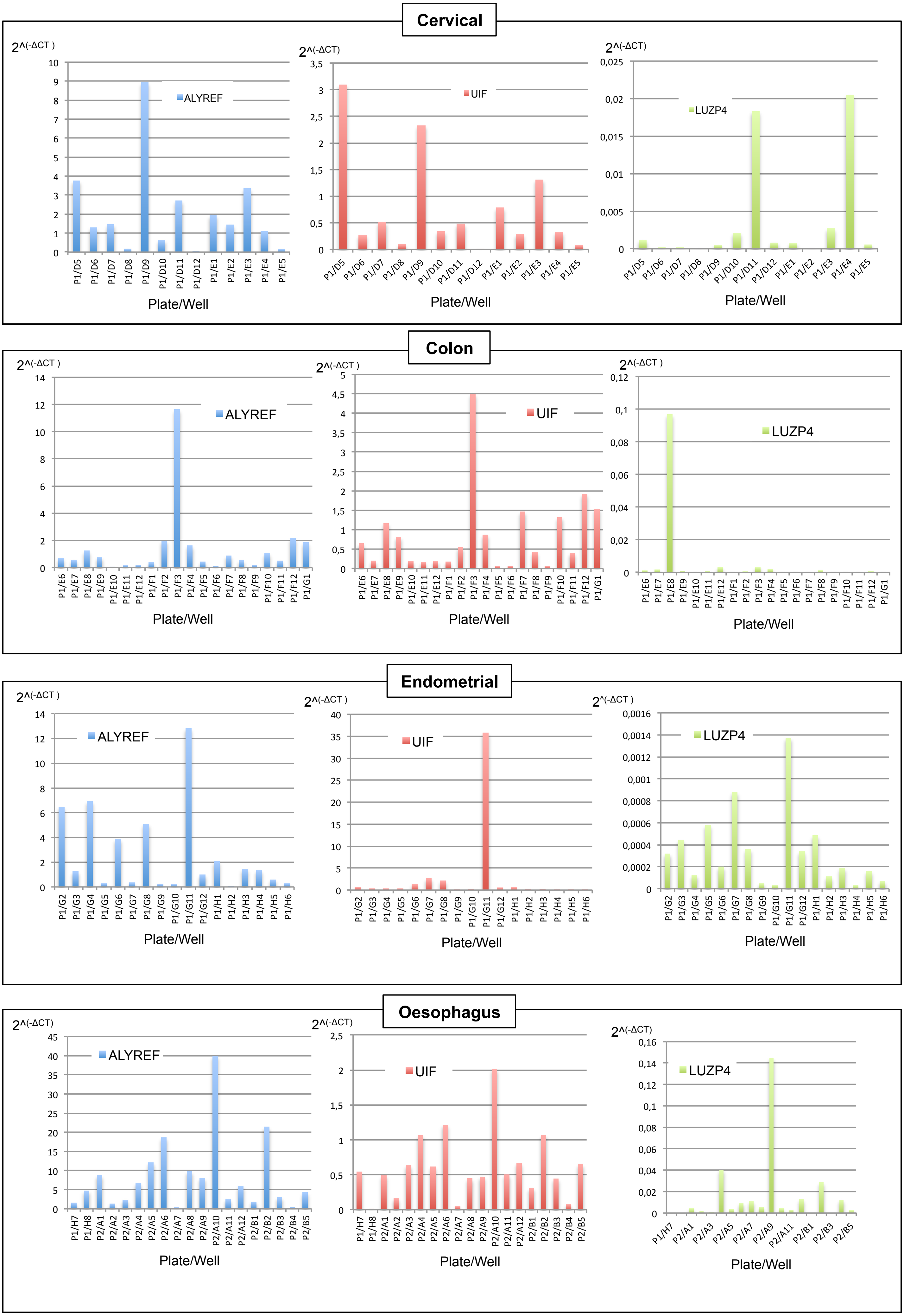

Cervical
2^(-ΔCT)
ALYREF
Plate/Well
2^(-ΔCT)
UIF
Plate/Well
2^(-ΔCT)
LUZP4
Plate/Well
Colon
2^(-ΔCT)
ALYREF
Plate/Well
2^(-ΔCT)
UIF
Plate/Well
2^(-ΔCT)
LUZP4
Plate/Well
Endometrial
2^(-ΔCT)
ALYREF
Plate/Well
2^(-ΔCT)
UIF
Plate/Well
2^(-ΔCT)
LUZP4
Plate/Well
Oesophagus
2^(-ΔCT)
ALYREF
Plate/Well
2^(-ΔCT)
UIF
Plate/Well
2^(-ΔCT)
LUZP4
Plate/Well

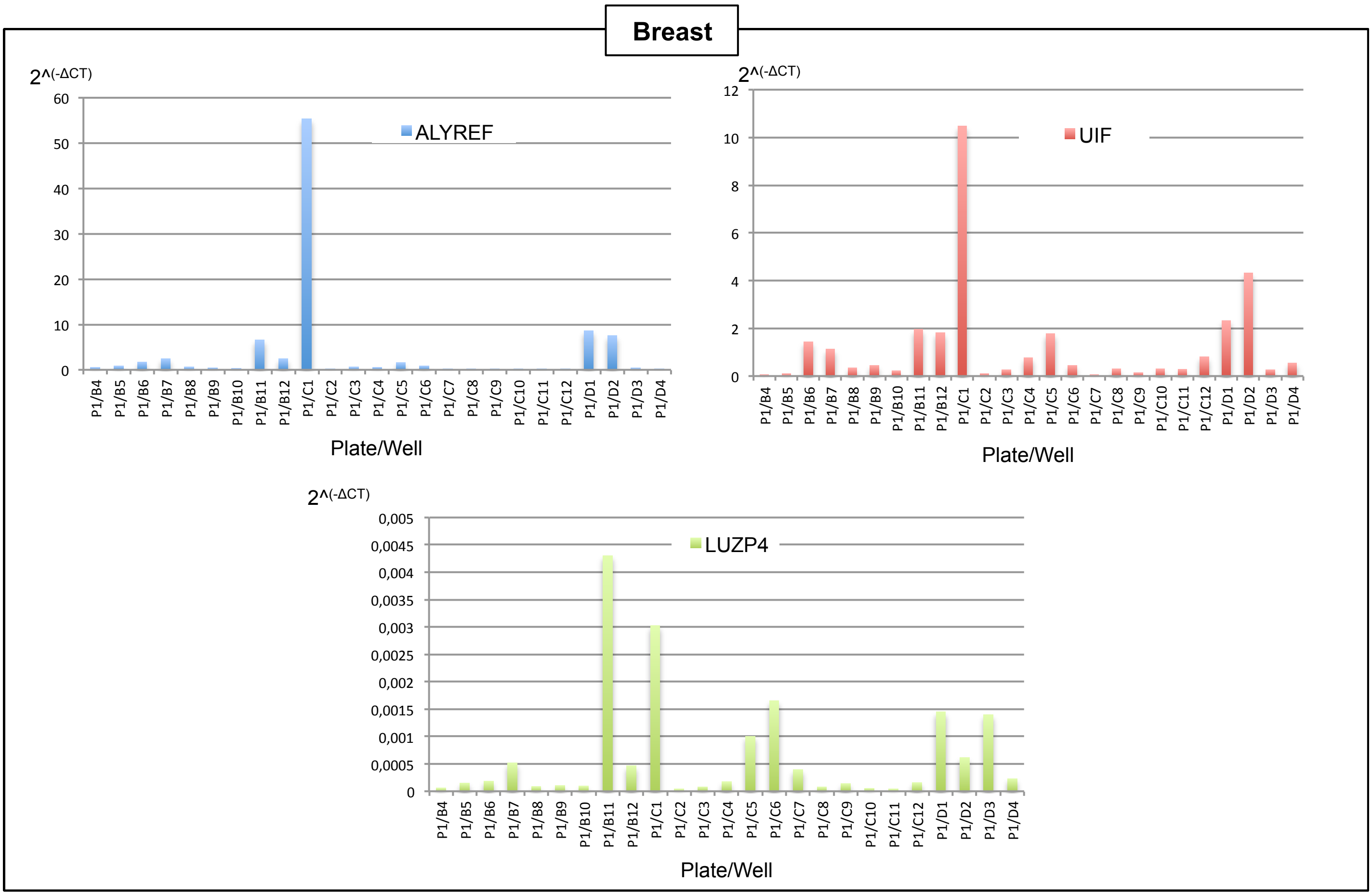
Breast
2^(-ΔCT)
ALYREF
Plate/Well
2^(-ΔCT)
UIF
Plate/Well
2^(-ΔCT)
LUZP4
Plate/Well

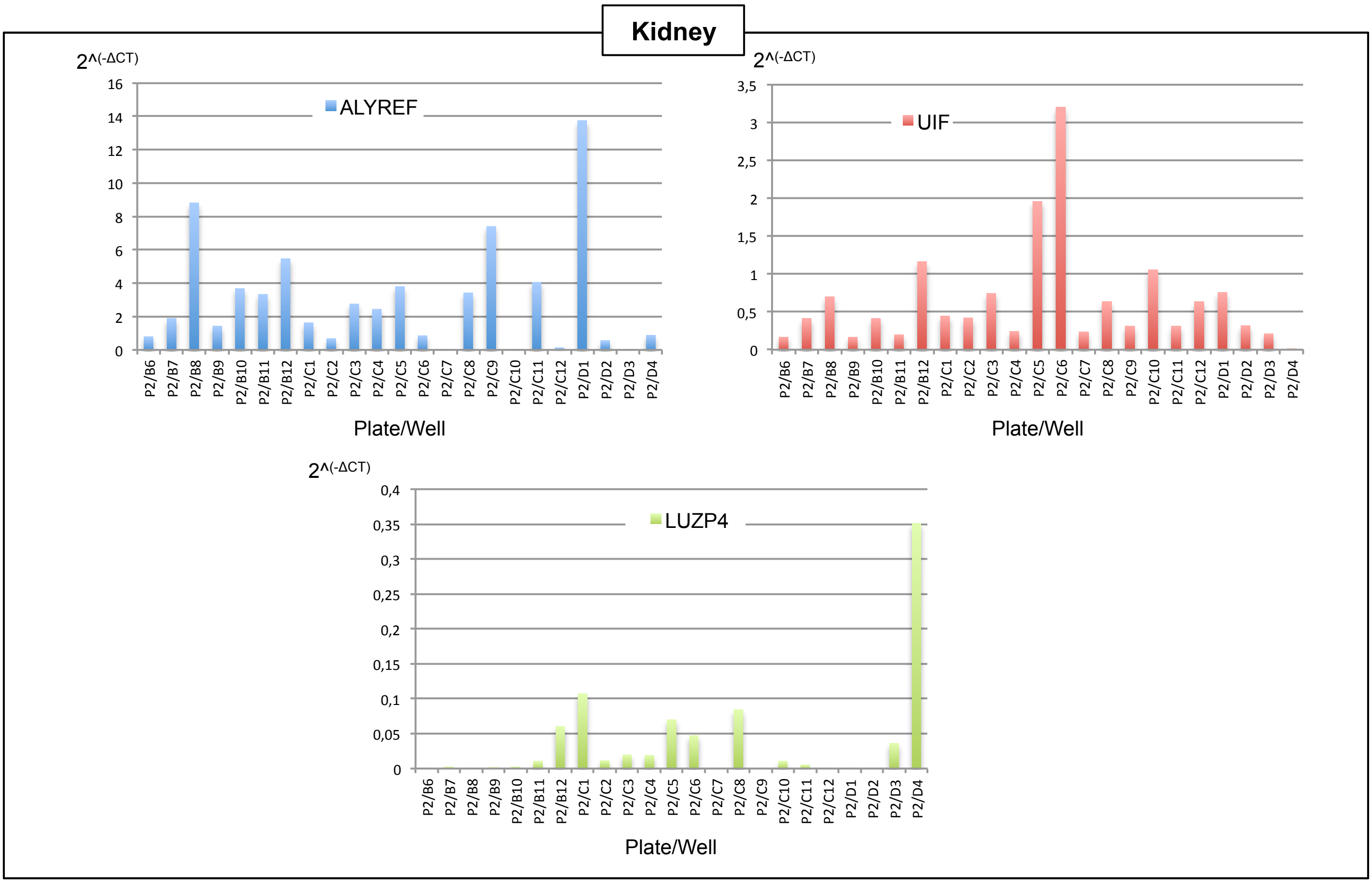
Kidney
2^(-ΔCT)
ALYREF
Plate/Well
2^(-ΔCT)
UIF
Plate/Well
2^(-ΔCT)
LUZP4
Plate/Well

Ovarian

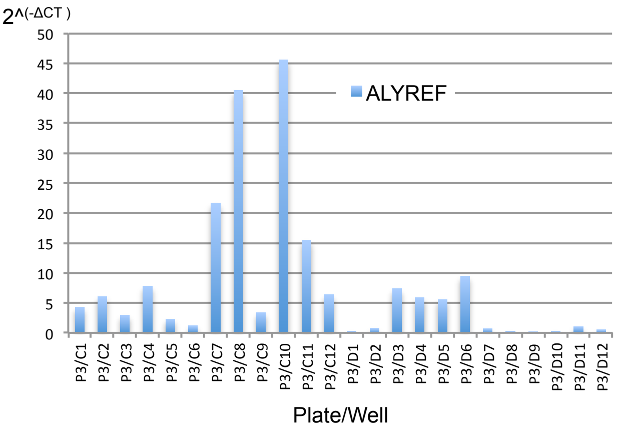

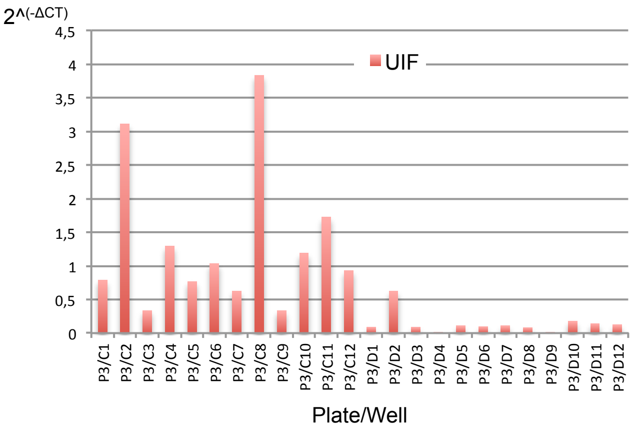

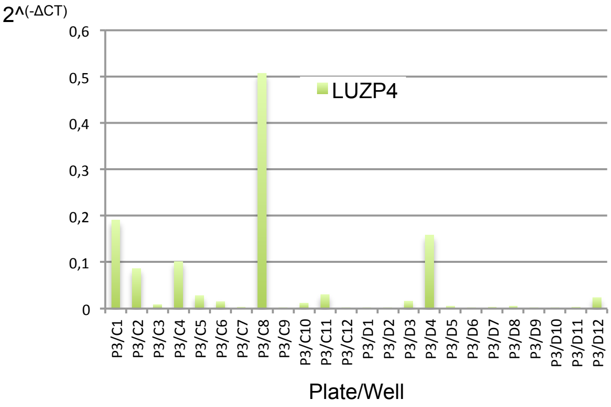

Lung

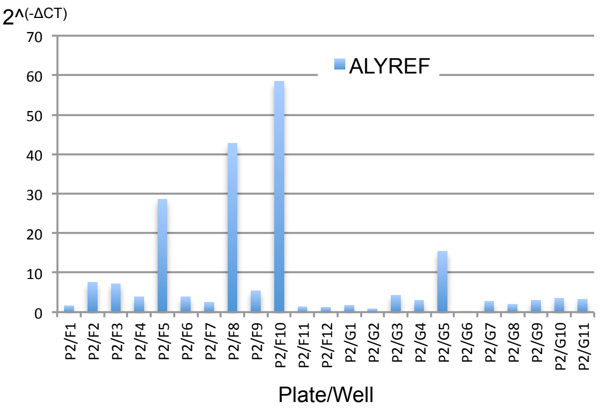

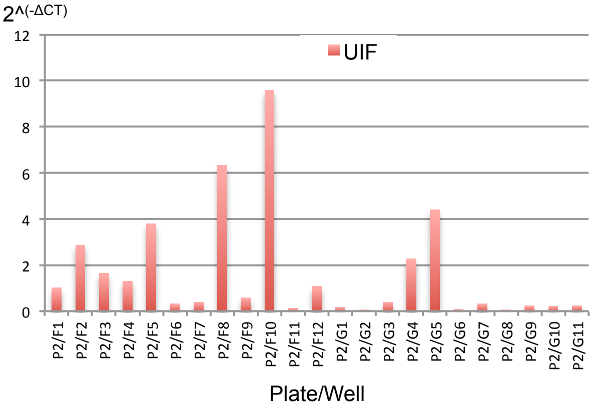

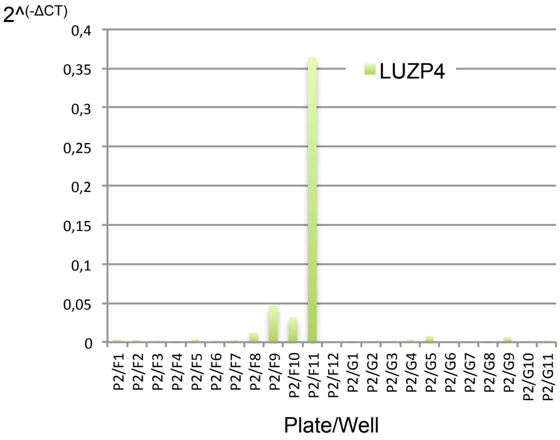

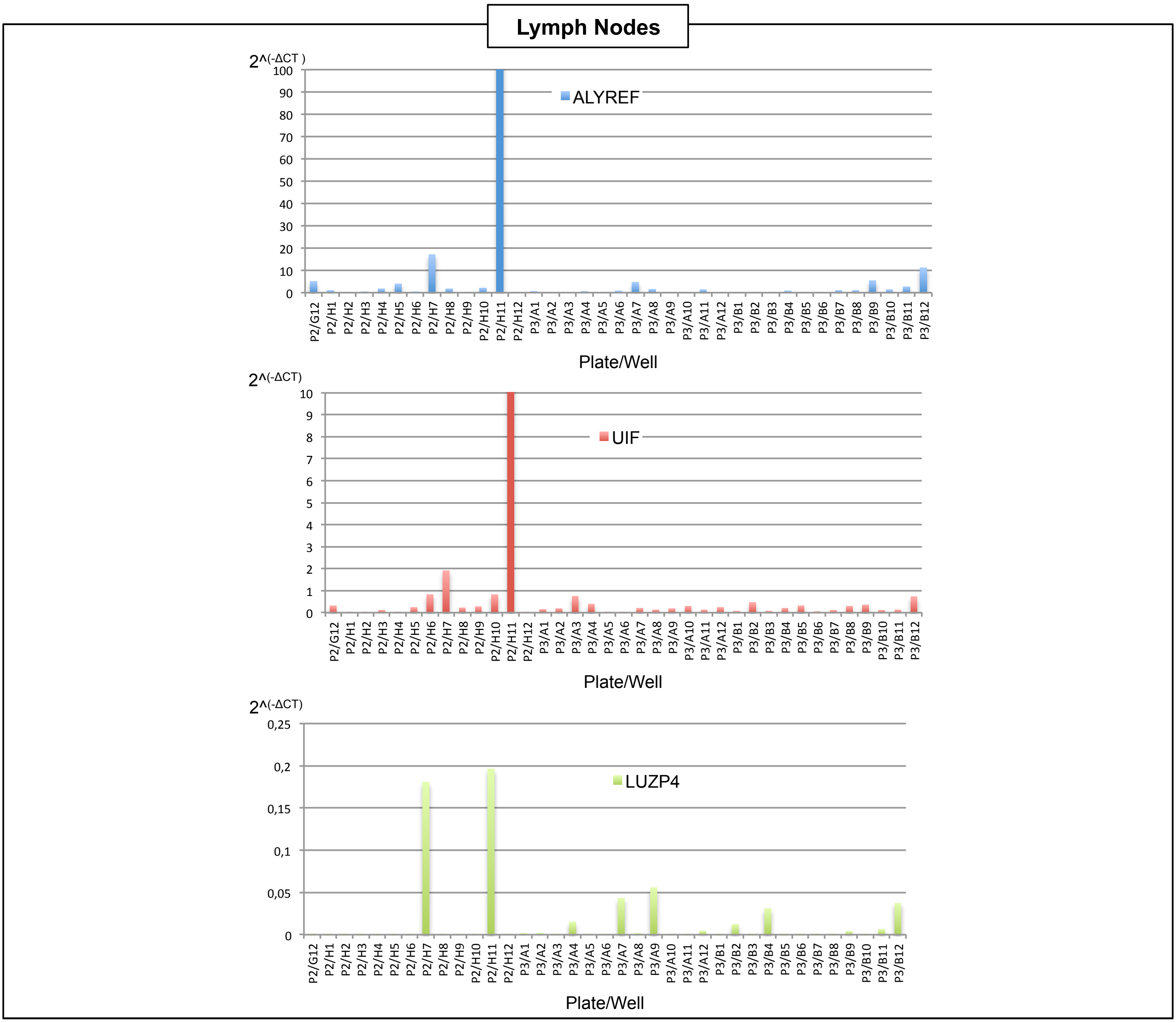

Lymph Nodes
2^(-ΔCT)
100
90
80
70
60
50
40
30
20
10
0
ALYREF
P2/G12 P2/H1 P2/H2 P2/H3 P2/H4 P2/H5 P2/H6 P2/H7 P2/H8 P2/H9 P2/H10 P2/H11 P2/H12 P3/A1 P3/A2 P3/A3 P3/A4 P3/A5 P3/A6 P3/A7 P3/A8 P3/A9 P3/A10 P3/A11 P3/A12 P3/B1 P3/B2 P3/B3 P3/B4 P3/B5 P3/B6 P3/B7 P3/B8 P3/B9 P3/B10 P3/B11 P3/B12
Plate/Well
2^(-ΔCT)
10
9
8
7
6
5
4
3
2
1
0
UIF
P2/G12 P2/H1 P2/H2 P2/H3 P2/H4 P2/H5 P2/H6 P2/H7 P2/H8 P2/H9 P2/H10 P2/H11 P2/H12 P3/A1 P3/A2 P3/A3 P3/A4 P3/A5 P3/A6 P3/A7 P3/A8 P3/A9 P3/A10 P3/A11 P3/A12 P3/B1 P3/B2 P3/B3 P3/B4 P3/B5 P3/B6 P3/B7 P3/B8 P3/B9 P3/B10 P3/B11 P3/B12
Plate/Well
2^(-ΔCT)
0,25
0,2
0,15
0,1
0,05
0
LUZP4
P2/G12 P2/H1 P2/H2 P2/H3 P2/H4 P2/H5 P2/H6 P2/H7 P2/H8 P2/H9 P2/H10 P2/H11 P2/H12 P3/A1 P3/A2 P3/A3 P3/A4 P3/A5 P3/A6 P3/A7 P3/A8 P3/A9 P3/A10 P3/A11 P3/A12 P3/B1 P3/B2 P3/B3 P3/B4 P3/B5 P3/B6 P3/B7 P3/B8 P3/B9 P3/B10 P3/B11 P3/B12
Plate/Well

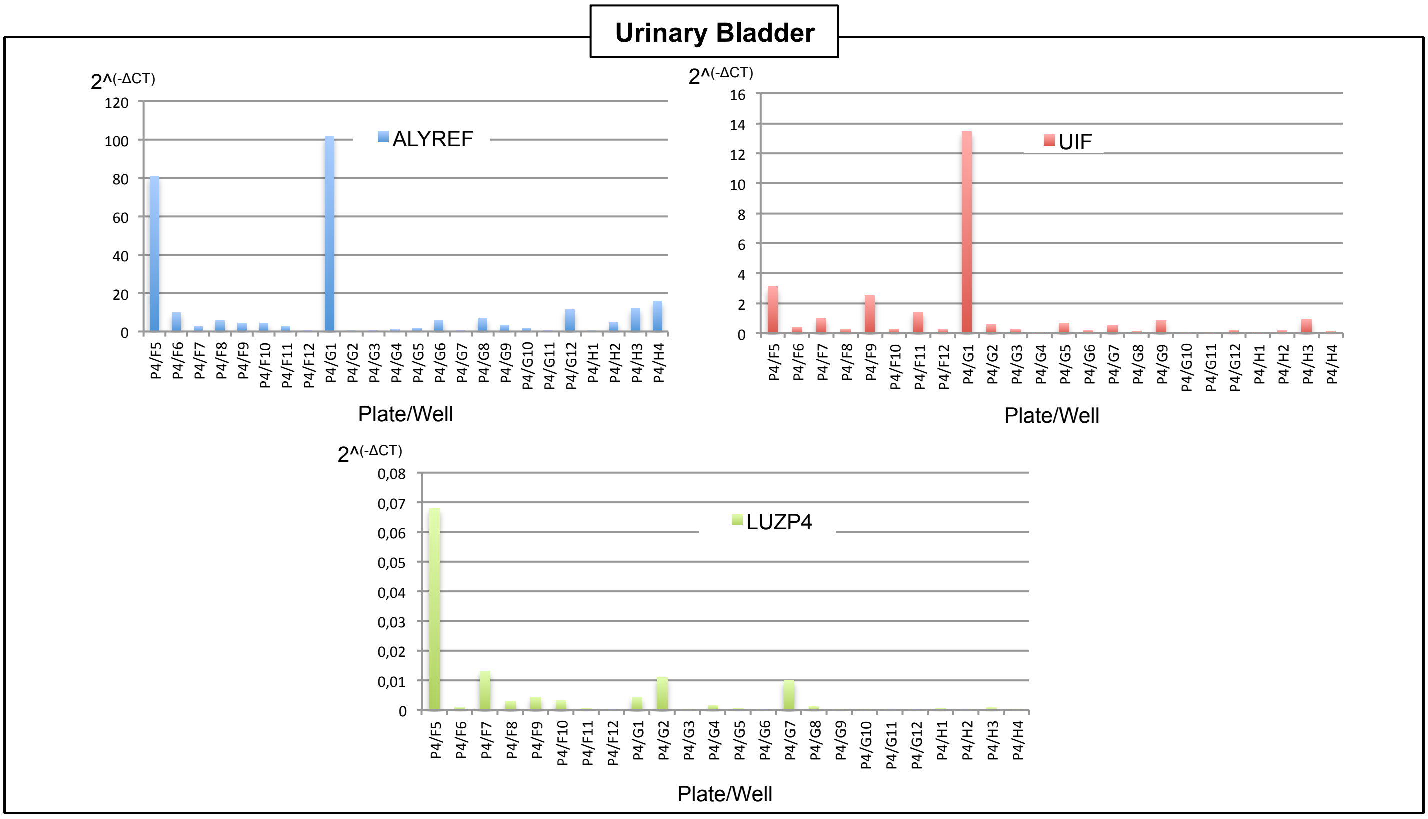

Urinary Bladder
2^(-ΔCT)
120
100
80
60
40
20
0
ALYREF
P4/F5 P4/F6 P4/F7 P4/F8 P4/F9 P4/F10 P4/F11 P4/F12 P4/G1 P4/G2 P4/G3 P4/G4 P4/G5 P4/G6 P4/G7 P4/G8 P4/G9 P4/G10 P4/G11 P4/G12 P4/H1 P4/H2 P4/H3 P4/H4
Plate/Well
2^(-ΔCT)
16
14
12
10
8
6
4
2
0
UIF
P4/F5 P4/F6 P4/F7 P4/F8 P4/F9 P4/F10 P4/F11 P4/F12 P4/G1 P4/G2 P4/G3 P4/G4 P4/G5 P4/G6 P4/G7 P4/G8 P4/G9 P4/G10 P4/G11 P4/G12 P4/H1 P4/H2 P4/H3 P4/H4
Plate/Well
2^(-ΔCT)
0,08
0,07
0,06
0,05
0,04
0,03
0,02
0,01
0
LUZP4
P4/F5 P4/F6 P4/F7 P4/F8 P4/F9 P4/F10 P4/F11 P4/F12 P4/G1 P4/G2 P4/G3 P4/G4 P4/G5 P4/G6 P4/G7 P4/G8 P4/G9 P4/G10 P4/G11 P4/G12 P4/H1 P4/H2 P4/H3 P4/H4
Plate/Well

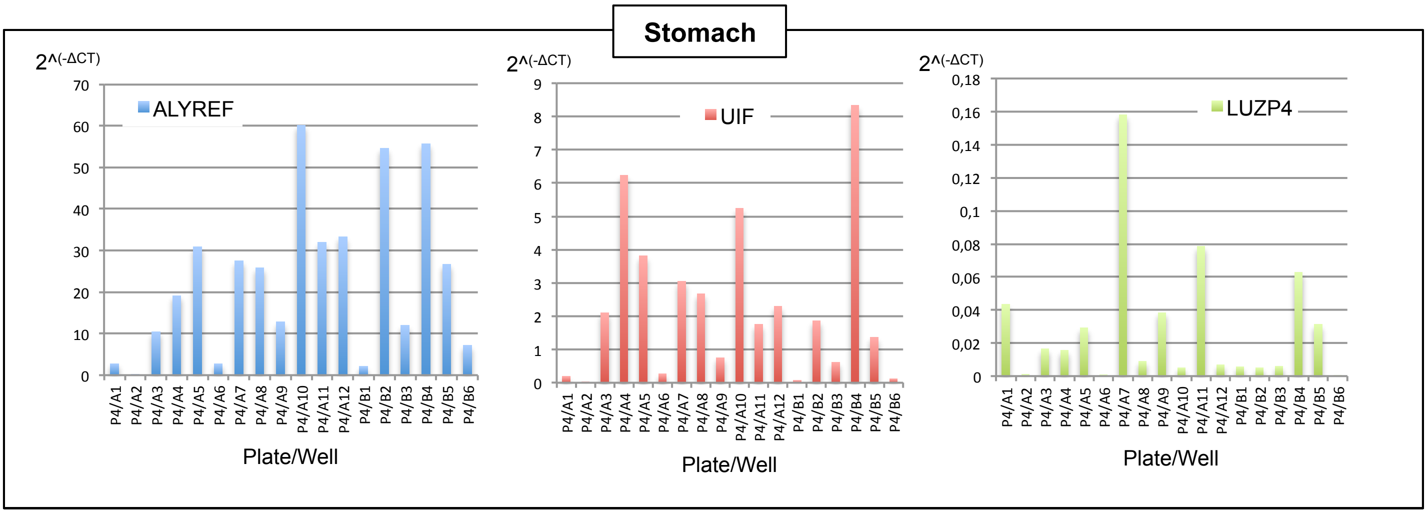
Stomach
2^(-ΔCT)
ALYREF
UIF
LUZP4
70 60 50 40 30 20 10 0
9 8 7 6 5 4 3 2 1 0
0,18 0,16 0,14 0,12 0,1 0,08 0,06 0,04 0,02 0
P4/A1 P4/A2 P4/A3 P4/A4 P4/A5 P4/A6 P4/A7 P4/A8 P4/A9 P4/A10 P4/A11 P4/A12 P4/B1 P4/B2 P4/B3 P4/B4 P4/B5 P4/B6
Plate/Well

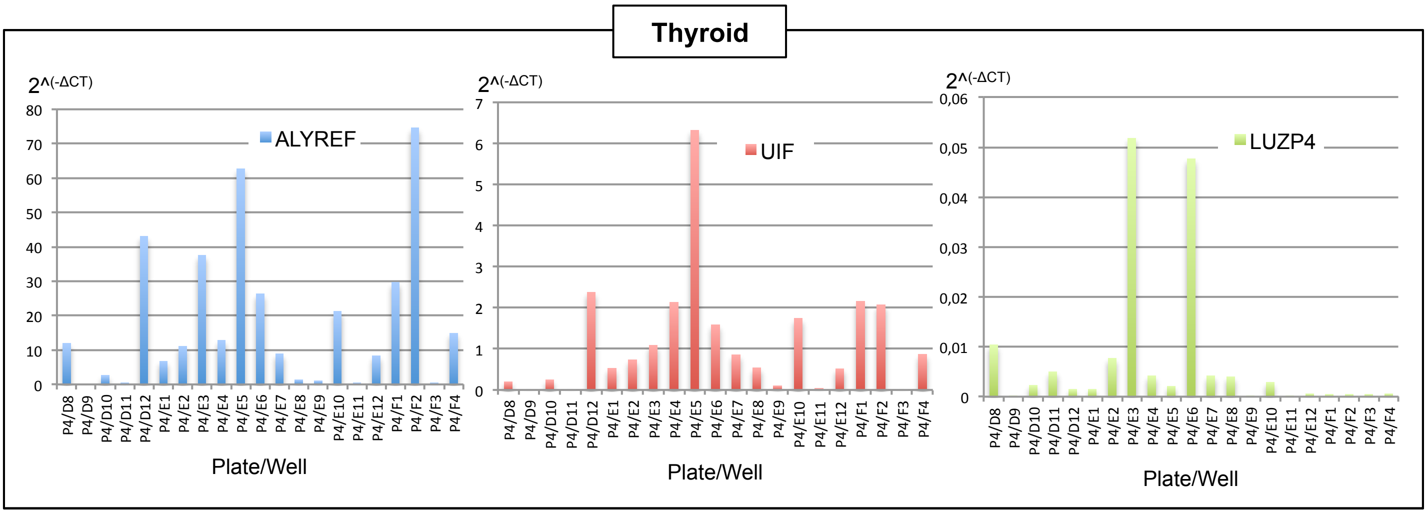
Thyroid
2^(-ΔCT)
ALYREF
UIF
LUZP4
80 70 60 50 40 30 20 10 0
7 6 5 4 3 2 1 0
0,06 0,05 0,04 0,03 0,02 0,01 0
P4/D8 P4/D9 P4/D10 P4/D11 P4/D12 P4/E1 P4/E2 P4/E3 P4/E4 P4/E5 P4/E6 P4/E7 P4/E8 P4/E9 P4/E10 P4/E11 P4/E12 P4/F1 P4/F2 P4/F3 P4/F4
Plate/Well

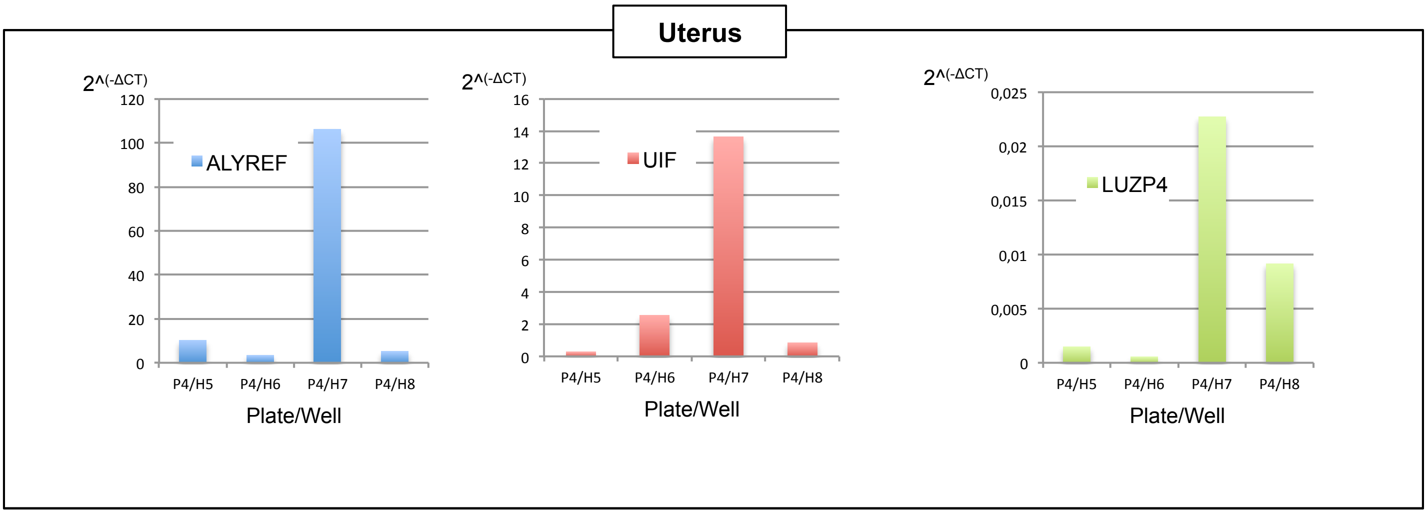
Uterus
2^(-ΔCT)
ALYREF
UIF
LUZP4
120 100 80 60 40 20 0
16 14 12 10 8 6 4 2 0
0,025 0,02 0,015 0,01 0,005 0
P4/H5 P4/H6 P4/H7 P4/H8
Plate/Well

Prostate

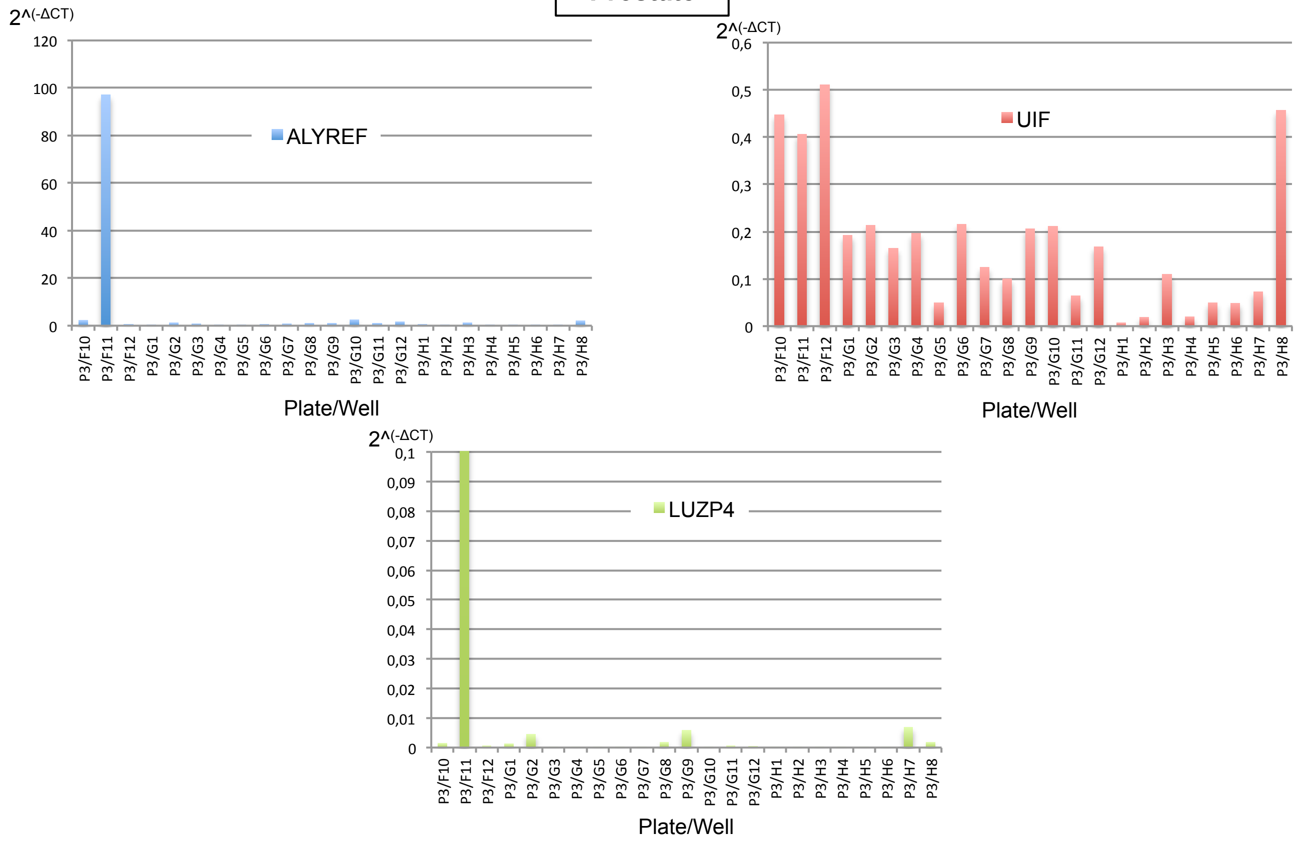

Skin

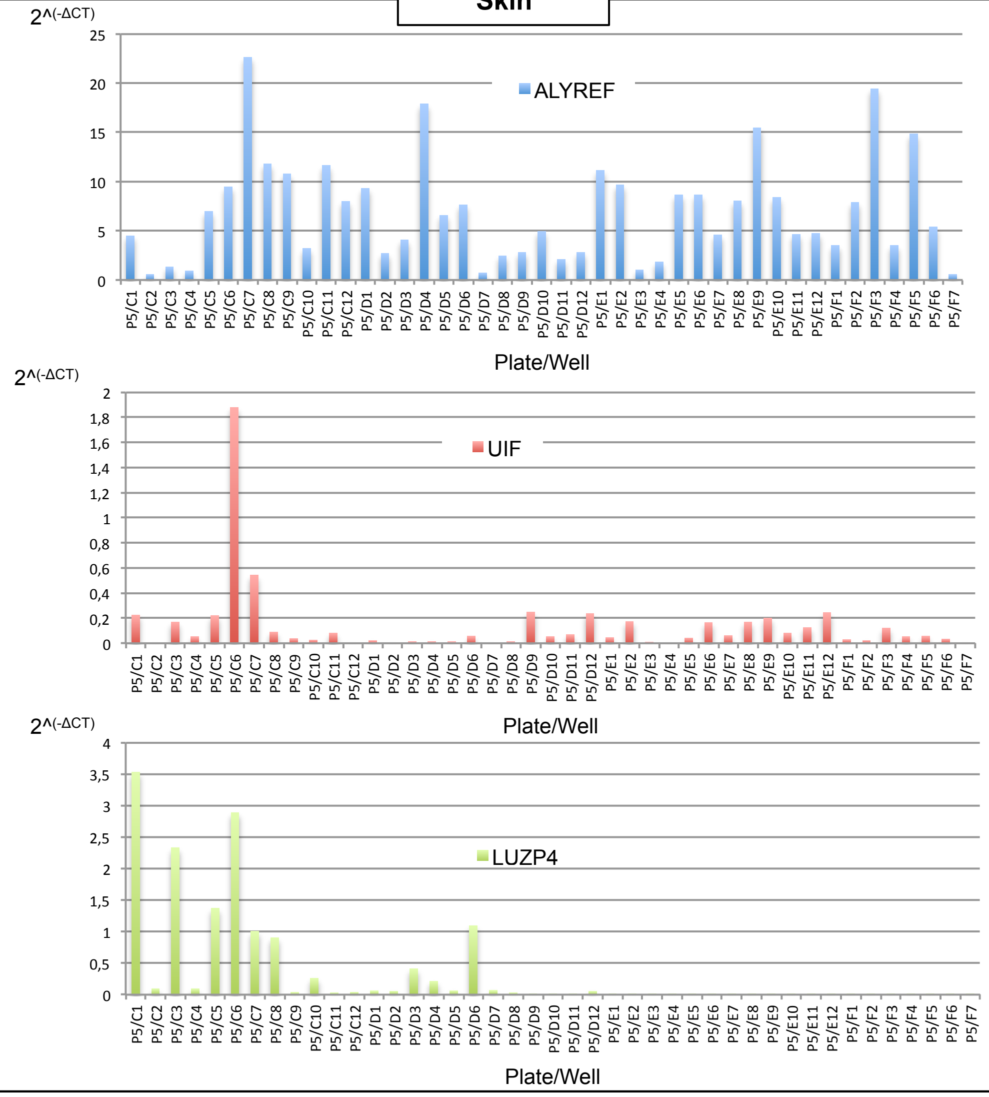

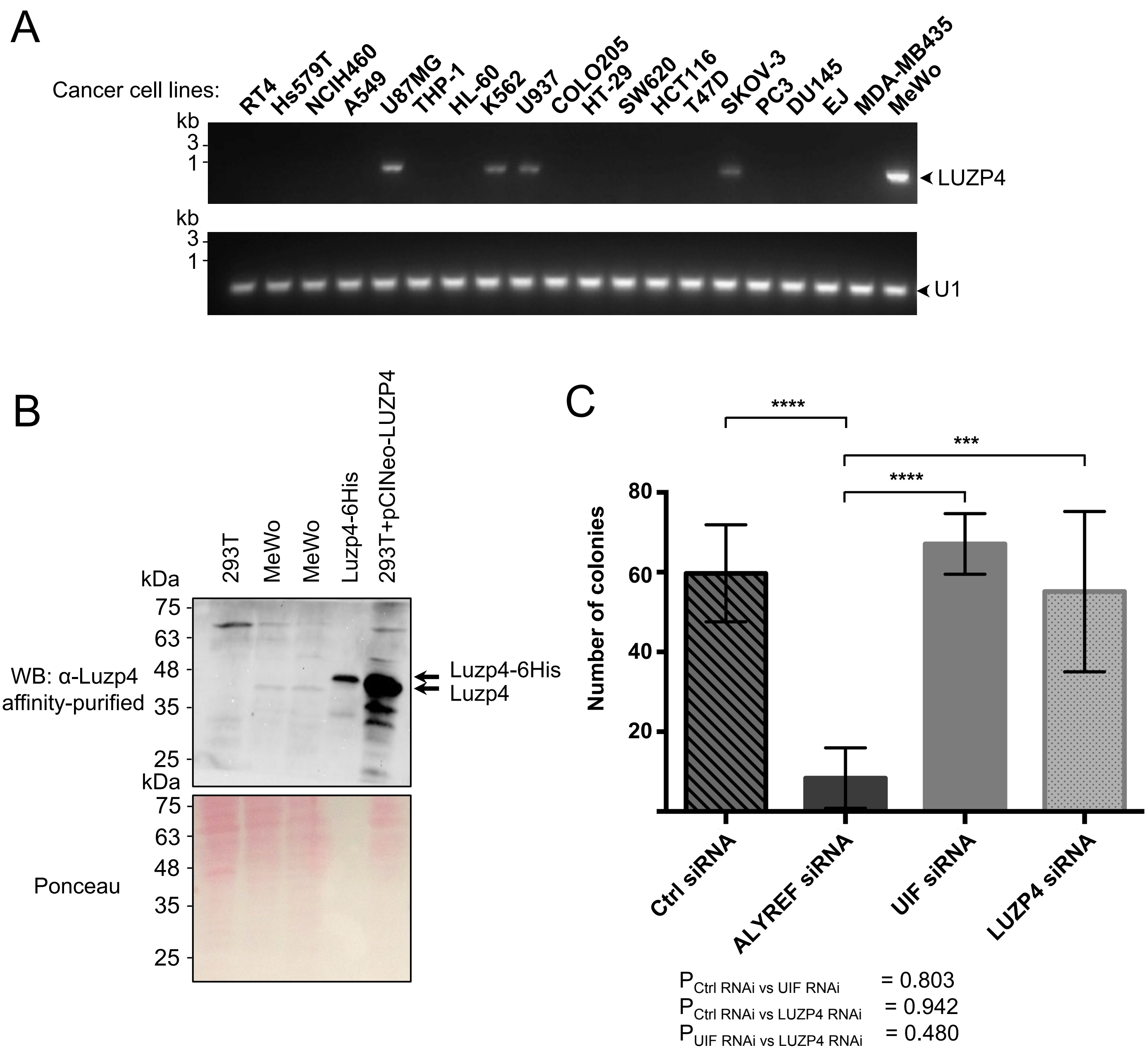

Supplementary Figure S8.

(A) Expression of Luzp4 in cancer cell lines. Various cancer cell lines were screened by non-saturating RT-PCR for LUZP4 expression and analysed by agarose gel electrophoresis. U1 RT-PCR was used to confirm efficient cDNA synthesis in each sample. LUZP4 RT-PCR primers were: forward 5'-GGAGAGGCTACTCAAGATGCAGAAGC-3' and reverse 5'-GAGATCACTCTGAGTGTCCACAAGATCTCC-3'. U1 RT-PCR primers were: forward 5'-ACCTGGCAGGGGAGATACCA-3' and reverse 5'-GGGGAAAGCGCGAACGCAGT-3'. **(B)** Western blot using our best anti-Luzp4 antibody, affinity purified, which recognizes recombinant or transfected Luzp4, but doesn't detect any endogenous Luzp4. 40 µg of cell extracts and 20 ng of recombinant Luzp4-6His. Affinity-purified anti-Luzp4 used at 1:500. **(C)** LUZP4 RNAi has no effect on 293T cells proliferation. Colony formation assays performed on 293T cells treated with the indicated siRNAs. Colonies that were scored contained >50 cells; mean values for five independent experiments are shown and error bars represent the standard deviation. Related to Fig.6D. *: $P<0.05$. **: $P<0.01$. ***: $P<0.001$. ****: $P<0.0001$.

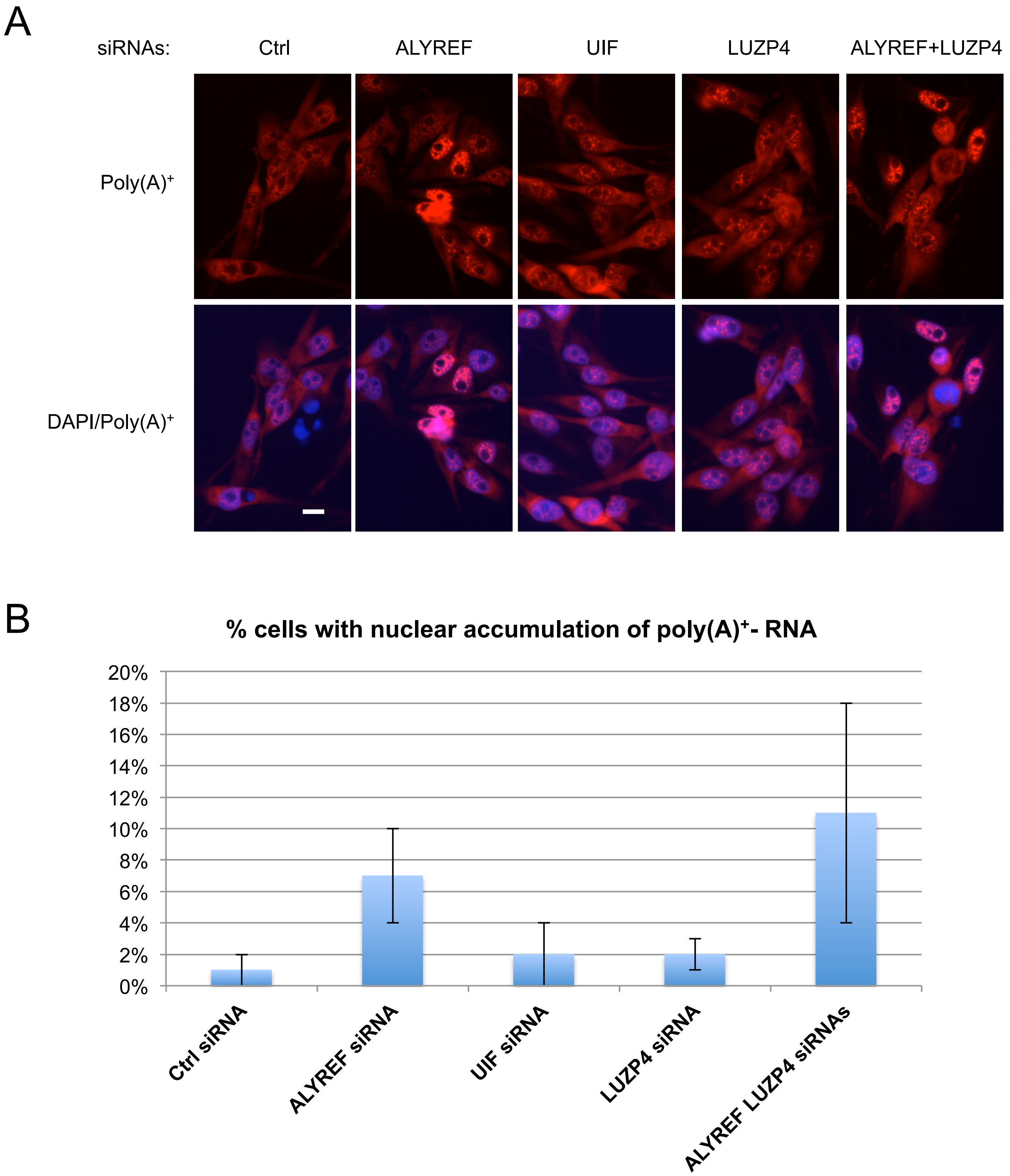

Supplementary Figure S9.

(A) Cy3-labeled oligo-$(dT)_{50}$ FISH was used to assess the localization of poly$(A)^+$-RNAs in MeWo cells treated with various siRNAs (top panels). An overlay of DAPI stained cells and the poly$(A)^+$ signal is shown in the bottom panels. Cells were siRNA-treated twice over a period of 72 hours. All equivalent panels are shown at the same exposure. The scale bar corresponds to 10 µm.

(B) Quantification for the results presented in panel A. Four images (with at least 60 cells each) per condition were used to count the proportion of cells displaying a nuclear accumulation of poly$(A)^+$ RNAs. Error bars represent the standard deviation.
